# Suppression of p16 alleviates the senescence-associated secretory phenotype

**DOI:** 10.18632/aging.202640

**Published:** 2021-02-06

**Authors:** Raquel Buj, Kelly E. Leon, Marlyn A. Anguelov, Katherine M. Aird

**Affiliations:** 1 Department of Pharmacology and Chemical Biology, UPMC Hillman Cancer Center, University of Pittsburgh School of Medicine, Pittsburgh, PA 15213, USA; 2 Biomedical Sciences Graduate Program, Penn State College of Medicine, Hershey, PA 17033, USA

**Keywords:** interleukin-6, interleukin-8, LMNB1, inflammation, melanoma

## Abstract

Oncogene-induced senescence (OIS) is characterized by increased expression of the cell cycle inhibitor p16, leading to a hallmark cell cycle arrest. Suppression of p16 in this context drives proliferation, senescence bypass, and contributes to tumorigenesis. OIS cells are also characterized by the expression and secretion of a widely variable group of factors collectively termed the senescence-associated secretory phenotype (SASP). The SASP can be both beneficial and detrimental and affects the microenvironment in a highly context-dependent manner. The relationship between p16 suppression and the SASP remains unclear. Here, we show that knockdown of p16 decreases expression of the SASP factors and pro-inflammatory cytokines *IL6* and *CXCL8* in multiple models, including OIS and DNA damage-induced senescence. Notably, this is uncoupled from the senescence-associated cell cycle arrest. Moreover, low p16 expression in both cancer cell lines and patient samples correspond to decreased SASP gene expression, suggesting this is a universal effect of loss of p16 expression. Together, our data suggest that p16 regulates SASP gene expression, which has implications for understanding how p16 modulates both the senescent and tumor microenvironment.

## INTRODUCTION

Senescence is considered a state of stable cell cycle arrest that can occur due to a variety of stimuli [1]. Oncogene-induced senescence (OIS) occurs upon activation of an oncogene such as HRAS or BRAF in normal cells [2, 3]. One of the hallmarks of senescent cells is upregulation of the cell cycle inhibitor *CDKN2A* (encoding for p16), which restrains cell cycle progression and cellular proliferation [4–6]. Canonically, elevated p16 represses hyperphosphorylation of the retinoblastoma protein (RB), which inhibits E2F transcription factor-mediated expression of proliferative genes [7]. Loss of p16 is a common event in human cancer that has been linked to senescence bypass, increased proliferation, and malignant transformation though both canonical and non-canonical (RB-independent) pathways [8–12].

The acquisition of a senescence-associated secretory phenotype (SASP) is also characteristic of senescent cells [13]. The SASP is composed of a variety of soluble signaling factors including pro-inflammatory cytokines, chemokines, and growth factors, as well as proteases, insoluble extracellular matrix proteins and non-protein components that are transcriptionally and translationally upregulated and secreted into the surrounding microenvironment by senescent cells [14–18]. Due to the impact that SASP can exert on cellular physiology, this program is tightly regulated at multiple levels. At the transcriptional level, several transcription factors (NF-κB, C/EBP-β) and upstream regulators (p38 MAPK, GATA4, p53, and ATM) have been described to either positively or negatively regulate SASP gene expression [16, 19–25]. The SASP is also regulated at both the epigenetic [26–32] and translational level [17, 33]. Recent publications suggest that the initiation of SASP gene transcription during OIS is likely due to loss of lamin B1 (*LMNB1*) and nuclear integrity [34, 35], leading to the accumulation of cytoplasmic chromatin fragments (CCFs) [36, 37]. CCFs activate the cytosolic DNA sensor cyclic guanosine monophosphate (GMP)-adenosine monophosphate (AMP) synthase (cGAS) that catalyzes the synthesis of the second messenger cyclic GMP-AMP (cGAMP) to bind and activate stimulator of interferon genes (STING), leading to NF-κB activation and cytokine transcription [36, 38, 39]. Therefore, changes in *LMNB1* expression are tightly linked to SASP gene transcription.

It is well documented that the SASP modifies the cellular microenvironment and alters neighboring cells, exerting a pleiotropic effect that is not fully understood [40]. On one hand, SASP factors contribute to wound-healing [41–43], normal development [44, 45], and have tumor suppressive effects through the recruitment of different immune cells to clear premalignant cells, a process termed senescence surveillance [46–48]. On the other hand, SASP factors can be pro-tumorigenic by sustaining proliferation, invasion, metastasis, and chemoresistance [49–53]. This paradoxical double role of SASP factors is highly dependent on both genetic background and SASP composition, which is known to be both variable and dynamic [54]. Different genetic backgrounds, cellular contexts, and/or senescence inducers allow for different SASP programs that can promote or inhibit tumorigenesis [55–57]. Interestingly, different SASP programs can also induce senescence in neighboring cells in a paracrine manner that in turn express a particular SASP program [55]. Thus, the final beneficial or detrimental net effect of the SASP is governed by multiple mechanisms that are not yet fully understood [58]. Characterizing whether different genetic backgrounds lead to different SASP programs may be critical to develop efficient and personalized regimens for cancer patients. As ~50% of all human tumors have low p16 expression [59], understanding its role in regulating the SASP has implications for a large subset of patients.

Here, we investigated the effect of p16 suppression on SASP gene expression. We found that knockdown of p16 leads to decreased *IL6* and *CXCL8* (encoding IL8) SASP gene expression in both HRAS^G12V^ and BRAF^V600E^ models of OIS. This was not due to increased *LMNB1* expression or loss of senescence markers, indicating that these changes were not due to inhibition of upstream signaling or a simple artifact due to reduced senescence. We confirmed these results in p16-wildtype melanoma cells upon knockdown of p16 and in DNA damage-induced senescence. Moreover, using publicly-available data, we found that low *CDKN2A* expression in patient tumors is associated with a decrease in specific SASP programs. Together, our results suggest that p16 may have a role in transcriptionally regulating SASP factors, which has implications for understanding how loss of p16 affects the senescent and tumor microenvironment.

## RESULTS

### Knockdown of p16 abrogates oncogene-induced *IL6* and *CXCL8* expression

**Figure 1:**
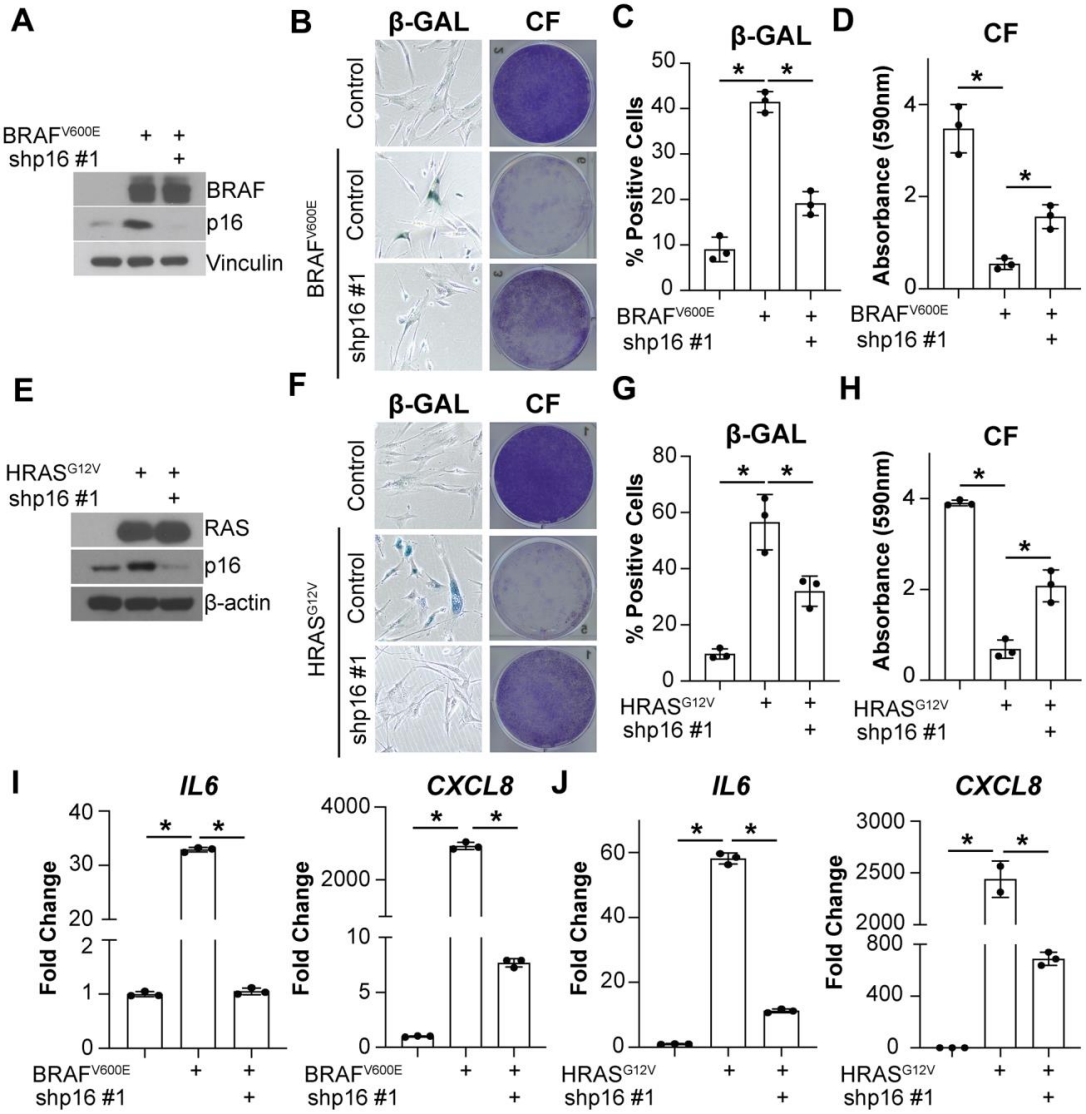
**Knockdown of p16 decreases IL6 and CXCL8 expression in oncogene-induced senescent cells.** IMR90s expressing either BRAF^V600E^ or HRAS^G12V^ alone or in combination with a shRNA targeting p16 (shp16 hairpin #1). An empty pBabe retroviral vector and a shRNA targeting GFP lentiviral vector were used as controls. See Supplementary Figure 1A for an experimental timeline. (**A**) Immunoblot of BRAF and p16. Vinculin was used a loading control. (**B**) Representative images of senescence-associated β-galactosidase (β-GAL) staining and colony formation (CF). (**C**) Quantification of β-GAL in (**B**). (**D**) Quantification of CF in (**B**). (**E**) Immunoblot of RAS and p16. β-actin was used as loading control. (**F**) Representative images of β-GAL staining and colony formation (CF). (**G**) Quantification of β-GAL in (**F**). (**H**) Quantification of CF in (**F**). (**I**, **J**) *IL6* and *CXCL8* mRNA expression (fold change relative to control mean). Expression of target genes was normalized against multiple reference genes. Data normalized against *MRPL9* are shown. n=3/group and mean±SD. 1 out of 3 experiments is shown. *p<0.05.

Upregulation of both p16 and SASP factors are characteristic of OIS cells [13]. A previous study found that overexpression of p16 induces senescence without upregulation of the SASP [60]. However, it is unknown whether p16 upregulation is necessary for SASP gene expression in the context of OIS. In order to better understand the effects of p16 expression on the SASP, we assessed the expression of the most extensively characterized interleukins upregulated in senescence, IL6 and CXCL8 [13, 16, 61]. Knockdown of p16 with BRAF^V600E^ or HRAS^G12V^ overexpression (Supplementary Figure 1A) decreased *IL6* and *CXCL8* expression and suppressed senescence-associated β-galactosidase (SA-β-gal) activity and the cell cycle arrest in IMR90 fibroblasts (Figure 1A–1J), a classical model of OIS [5, 62]. Note that abrogation of p16 expression using these conditions occurs prior to the induction of senescence that typically occurs 2-3 days after oncogene activation [5, 63–65]. A second shRNA targeting p16 confirmed the results, suggesting these observations are not due to off-target effects (Supplementary Figure 1B–1F). Similar results were observed in normal skin fibroblasts Hs 895.Sk (Supplementary Figure 1G, 1H), suggesting this is not a cell line-specific phenomenon. Additionally, knockdown of p16 in the BRAF^V600E^-induced senescence model decreased the expression of other SASP factors including growth factors, proteases, and ligands (Supplementary Figure 1I), suggesting that this is a broader phenomenon not limited to *IL6* and *CXCL8*. Together, these data suggest that knockdown of p16 may regulate the expression of multiple SASP factors upon oncogene activation.

**Figure 2:**
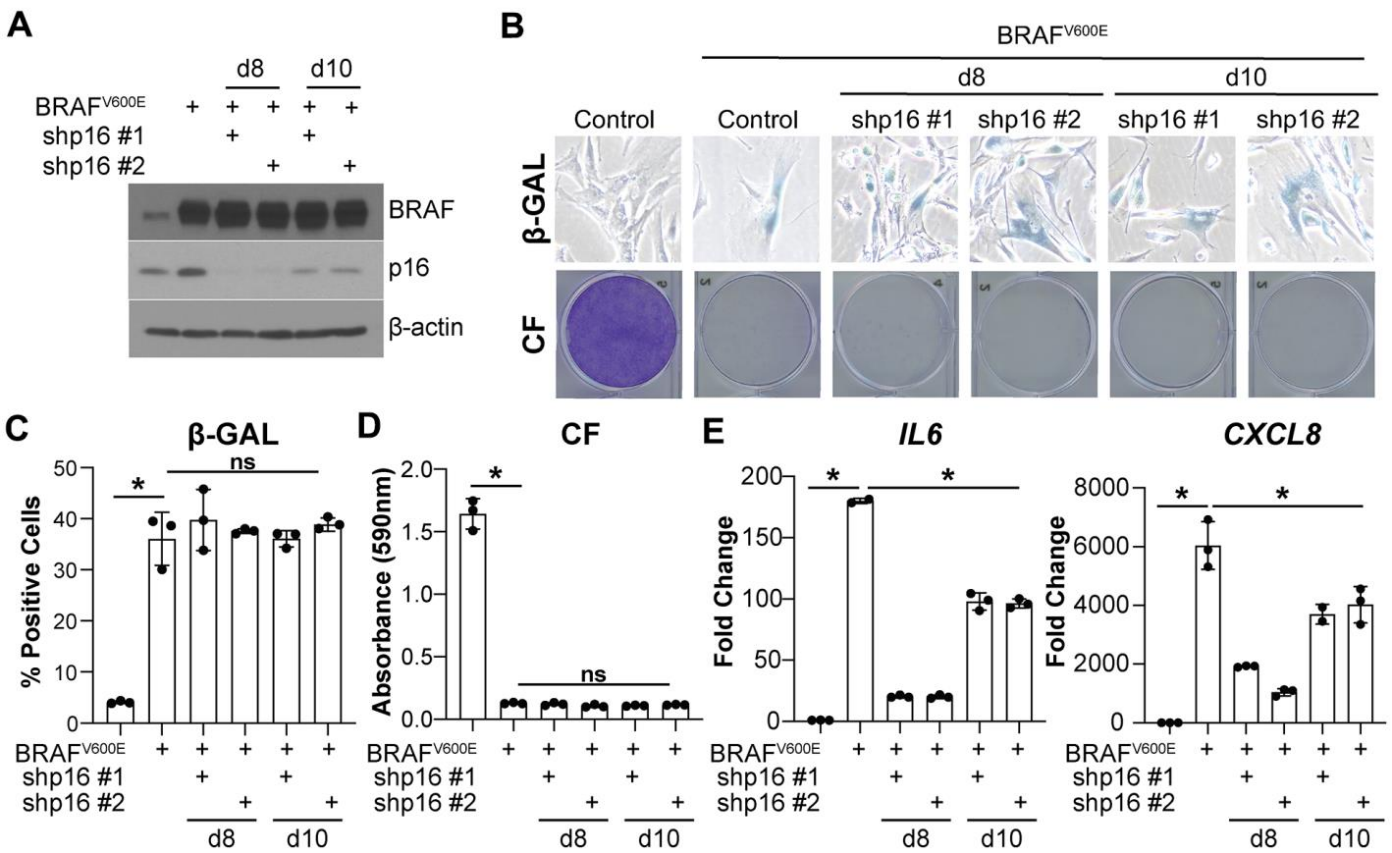
**Knockdown of p16 at later timepoints decreases IL6 and CXCL8 expression without bypassing oncogene-induced senescence.** IMR90s expressing BRAF^V600E^ alone or in combination with shRNAs targeting p16 (shp16 hairpin #1 and #2). An empty pBabe retroviral vector and a shRNA targeting GFP lentiviral vector were used as controls. See Supplementary Figure 2A for an experimental timeline. (**A**) Immunoblot of BRAF and p16. β-actin was used as loading control. (**B**) Representative images of senescence-associated β-galactosidase (β-GAL) staining and colony formation (CF). (**C**) Quantification of β-GAL in (**B**). (**D**) Quantification of CF in (**B**). (**E**) *IL6* and *CXCL8* mRNA expression (fold change relative to control mean). Expression of target genes was normalized against multiple reference genes. Data normalized against *PMSC4* are shown. n=3/group and mean±SD. 1 out of 3 experiments is shown. *p<0.05. ns = not significant.

Knockdown of p16 prior to the induction of BRAF^V600E^/HRAS^G12V^-mediated senescence bypasses the senescence-associated cell cycle arrest (Figure 1A–1H and Supplementary Figure 1B–1E) [8]. Therefore, it is possible that the SASP expression is low because the cells never undergo OIS. To investigate whether the observed decrease in *IL6* and *CXCL8* is a direct effect of p16 suppression and not simply a consequence of senescence bypass, we knocked down p16 at two time points after oncogene expression (Figure 2A and Supplementary Figure 2A–2C). Suppression of p16 at day 8 and 10 after oncogene expression did not bypass senescence as observed using multiple markers of senescence (Figure 2B–2D and Supplementary Figure 2D–2G). Consistent with observations using knockdown of p16 prior to senescence induction (Figure 1), *IL6* and *CXCL8* expression were both decreased when p16 was knocked down at later time points (Figure 2E and Supplementary Figure 2H). All together, these data suggest that the observed effects of p16 expression on *IL6* and *CXCL8* are uncoupled from senescence bypass.

### Knockdown of p16 in tumor cells decreases *IL6* and *CXCL8* expression

**Figure 3:**
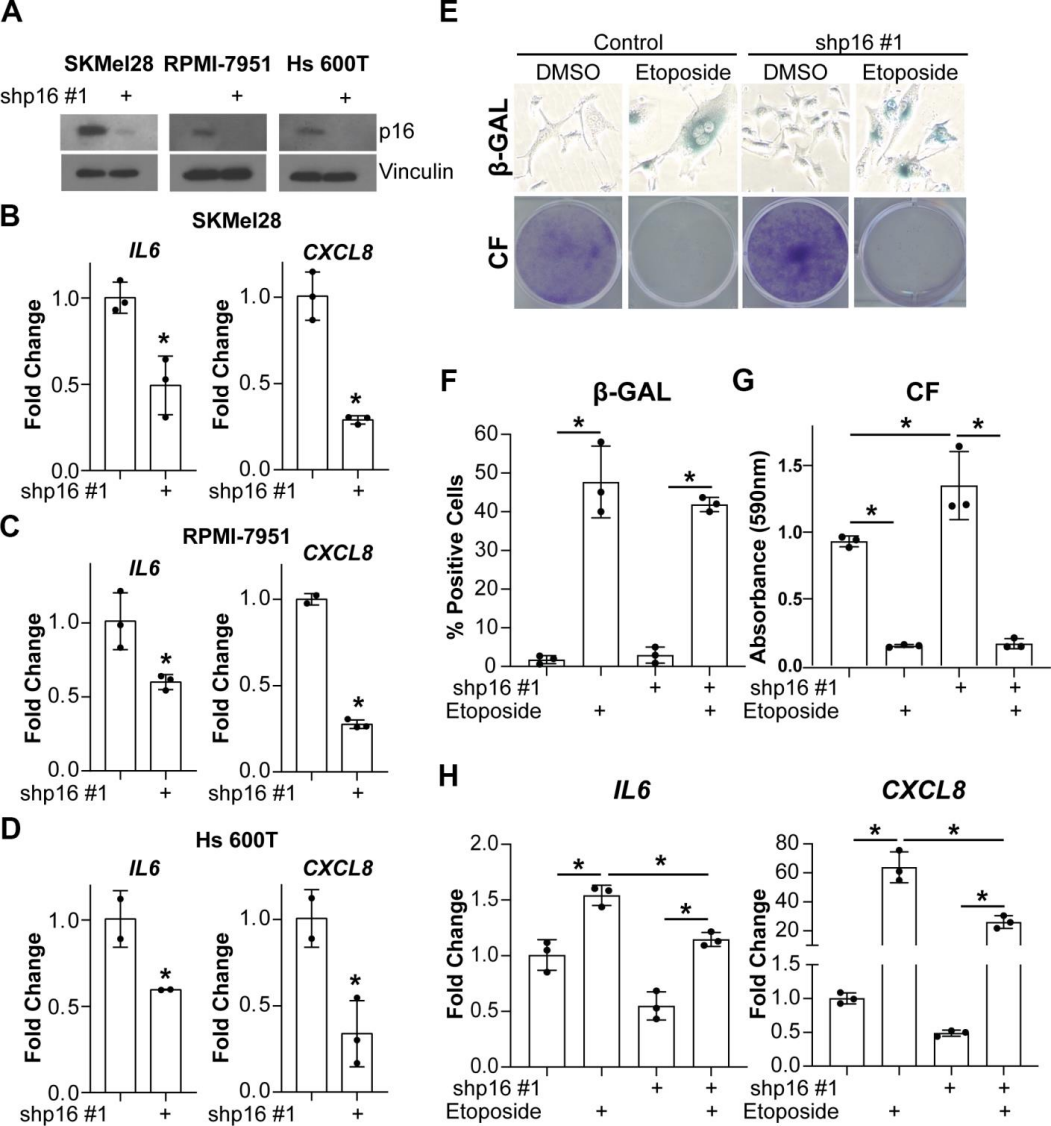
**Knockdown of p16 in melanoma cells decreases IL6 and CXCL8 expression.** The melanoma cell lines SKMel28, RPMI-7951, and Hs 600T expressing wildtype p16 were infected with lentivirus expressing a shRNA targeting p16 (shp16 hairpin #1). An shRNA targeting GFP lentiviral vector was used as control. (**A**) Immunoblot of p16. Vinculin was used as loading control. (**B**–**D**) mRNA expression of *IL6* and *CXCL8* (fold change relative to control mean) in SKMel28 (**B**), RPMI-7951 (**C**), and Hs 600T (**D**) melanoma cells. Expression of target genes was normalized against multiple reference genes. Data normalized against *MRPL9* are shown. n=3/group and mean±SD. 1 out of 3 experiments is shown. (**E**–**H**) p16 was stably knocked down in SKMel28 melanoma cells with a shRNA (shp16 hairpin #1). An shRNA targeting GFP lentiviral vector was used as control. Cells were treated with 1μM etoposide for 6 days. (**E**) Representative images of senescence-associated β-galactosidase (β-GAL) staining and colony formation (CF). (**F**) Quantification of β-GAL in (**E**). (**G**) Quantification of CF in (**E**). (**H**) *IL6* and *CXCL8* mRNA expression (fold change relative to control mean). Expression of target genes was normalized against multiple reference genes. Data normalized against *PMSC4* are shown. n=3/group and mean±SD. 1 out of 2 experiments is shown. *p<0.05.

Next, we aimed to investigate whether suppression of p16 leads to decreased expression of *IL6* and *CXCL8* in tumor cells. Towards this goal, we knocked down p16 in three melanoma cell lines with wildtype p16 (Figure 3A). Consistent with our data in fibroblasts (Figures 1, 2), knockdown of p16 in the melanoma cells also decreased *IL6* and *CXCL8* (Figure 3B–3D). Notably, this was not a consequence of a reduced burden of spontaneous senescent cells in p16 knockdown cells (Supplementary Figure 3A, 3B). Finally, expression of *IL6* and *CXCL8* was also significantly reduced by stable knockdown of p16 in melanoma cells induced to senesce using etoposide (Figure 3E–3H). Etoposide induced senescence to a similar extent in both p16 wildtype controls and p16 knockdown cells, suggesting the decrease in expression was not linked to decreased senescence (Figure 3E–3G). Altogether, these data suggest that p16 may directly or indirectly regulate the transcription of both *IL6* and *CXCL8* and support the hypothesis that this is not a consequence of p16 suppression-mediated senescence bypass since suppression of p16 in both senescent and proliferating melanoma cells abrogates *IL6* and *CXCL8*.

### Low *CDKN2A* in patient tumors correlates with low SASP expression

**Figure 4:**
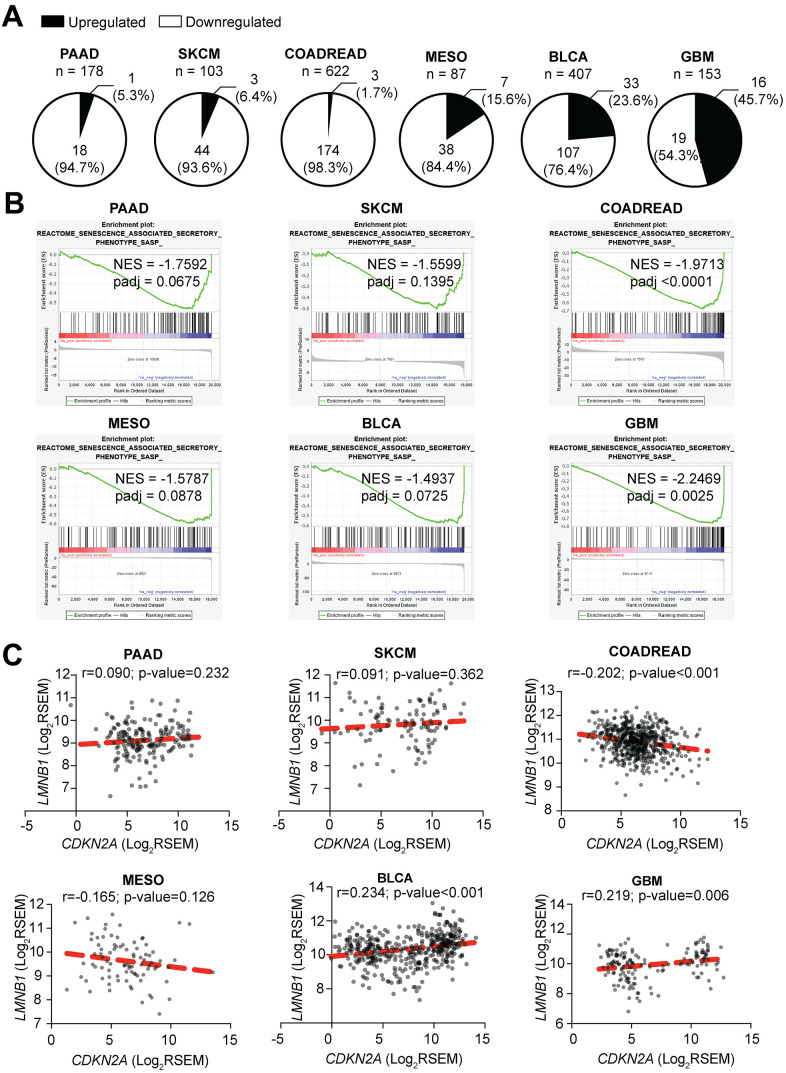
**Tumors with low CDKN2A expression have decreased expression of SASP.** (**A**) Percentage of SASP genes significantly upregulated and downregulated in *CDKN2A*-low (i.e., p16-low) expressing tumors when compared to *CDKN2A*-high (i.e., p16-high) expressing tumors. (**B**) Negatively enriched SASP term among the six studied tumor types in Gene Set Enrichment Analysis (GSEA) between *CDKN2A*-low and *CDKN2A*-high expressing tumors. SKCM (skin cutaneous melanoma), PAAD (pancreatic adenocarcinoma), COADREAD (colorectal adenocarcinoma), MESO (mesothelioma), BLCA (bladder urothelial carcinoma), GBM (glioblastoma multiforme), NES (negative enrichment score). (**C**) Correlation between *CDKN2A* and *LMNB1* expression for each tumor type. Data are shown as Log_2_ of RSEM. Coefficient of correlation (r) and p-value were calculated using Pearson’s correlation.

It has been widely demonstrated that suppression of p16 leads to increased proliferation, tumorigenesis, and metastasis *in vitro* and *in vivo*, and loss of p16 expression is considered a poor prognostic maker [66–69]. To further understand the relationship between loss of p16 and decreased expression of the SASP, we used TCGA data from primary tumors of skin cutaneous melanoma (SKCM, n=103), pancreatic adenocarcinoma (PAAD, n=178), colorectal adenocarcinoma (COADREAD, n=622), mesothelioma (MESO, n=87), bladder urothelial carcinoma (BLCA, n=407) and glioblastoma multiforme (GBM, n=153), six tumor types where loss of p16 is frequently observed and has clinical implications [70–77]. Patients were classified according to their *CDKN2A* status (p16-low or p16-high, see Methods for details) (Table 1), and differential expression analysis was performed independently for each tumor type. Note that there were no significant differences in the number of normal and tumor cells between p16-high and p16-low tumors (Supplementary Figure 4A). Most of the SASP factors profiled in a published database of RAS-induced senescence (including soluble factors and exosomes, 232 total unique genes) (Supplementary Table 1) [54], were significantly downregulated in p16-low tumors (Supplementary Figure 4A and Supplementary Table 2). Gene Set Enrichment Analysis (GSEA) also showed a decrease in ‘Senescence Associated Secretory Phenotype, SASP’ (Figure 4B) in p16-low tumors. Interestingly, 4 out of 6 tumors (PAAD, COADREAD, MESO, and BLCA) showed a decrease in pathways related to inflammation and the immune system such as ‘Antigen Processing and Presentation’ and ‘Cytosolic DNA Sensing Pathway’ in p16-low tumors (Supplementary Figure 4B). These data demonstrate that p16 status correlates with expression of SASP factors in human tumor samples. Importantly, to rule out the possibility of less senescent tumor cells in the p16-low patient samples, we used GSEA to cross-compare p16-low vs. p16-high expression profiles with a previously published senescence expression signature [78]. Among the 6 tumor types, only MESO showed a significant negative normalized enrichment score (NES) (Supplementary Table 3). This suggests that in 5 of the 6 tumor types, there was not a significant decrease in the number of intratumoral-senescent cells. Thus, the observed decrease in SASP expression signatures is likely not due to less intratumoral senescent cells. To unravel whether this observation is due to a decrease of immune cell infiltration in p16-low vs. p16-high tumors, we compared the number of infiltrating lymphocytes, monocytes, and neutrophils seen on OCT-embedded tissue slides reported by TCGA in 5 out of 6 tumors (note that there is no available data for GBM). No significant differences were observed between p16-low and p16-high tumors (Supplementary Figure 4C). These data at least in part suggest that changes in SASP expression are not due to differences in intratumoral senescent cells or differential infiltration of immune cells that could potentially bias our analysis.

**Table 1:** Statistics of TCGA data sets.

	**SKCM**	**PAAD**	**COADREAD**	**MESO**	**BLCA**	**GBM**
**Total cases**	103	178	622	87	407	153
***CDKN2A* expression (Log**_2_**RSEM) mean**	7.08	6.73	6.31	6.25	7.56	6.61
***CDKN2A* expression (Log**_2_**RSEM) standard deviation (SD)**	2.76	2.32	1.74	2.26	3.61	3.15
***CDKN2A* expression (Log**_2_**RSEM) median**	7.75	6.35	6.22	6.29	8.22	5.11
***CDKN2A* expression (Log**_2_**RSEM) first quartile (Q**_1_**)**	5.15	4.93	5.20	4.59	4.16	4.07
***CDKN2A* expression (Log**_2_**RSEM) third quartile (Q**_3_**)**	9.02	8.38	7.30	7.68	10.82	10.40
**No. p16-low cases (*CDKN2A* expression ≤ Q**_1_**)**	103	45	156	22	102	38
**No. p16-high cases (*CDKN2A* expression ≥ Q**_3_**)**	103	45	156	22	102	38

Finally, since we observed a downregulation of the “Cytosolic DNA Sensing Pathway” signature, we correlated the expression level of *CDKN2A* and *LMNB1* in the tumors. We did not observe a strong correlation between *CDKN2A* and *LMNB1* expression (Figure 4C), suggesting that p16 regulation of this pathway and the SASP in tumors may not be directly through modulation of *LMNB1*. However, we cannot rule out the possibility that other pathways may be altered to affect the cytosolic DNA sensing pathway. All together with our *in vitro* data, these data in human tumor samples demonstrate a universal, positive correlation between p16 expression and SASP gene expression.

## DISCUSSION

Increased expression of p16 and the SASP are characteristics of OIS; however, the relationship between them is not well understood. While increased p16 expression has a clear role in sustaining the characteristic cell cycle arrest of OIS cells [8, 79], the SASP appears to be a consequence of the DNA damage response induced by OIS and is not necessary for the proliferative arrest [24, 80, 81]. Indeed, this is evident by the observation that p16-mediated senescence induction, a stimulus that a priori only restrains cell cycle progression, does not induce SASP expression [60]. Here we found that knockdown p16 decreases gene expression of two of the most well characterized SASP factors *IL6* and *CXCL8* [13, 16, 61], in a manner that is uncoupled from senescence bypass (Figures 1, 2). Knockdown of p16 or low expression of *CDKN2A* in patient tumors was also associated with lower expression of additional SASP factors. Together, this suggests that p16 expression is not sufficient but is necessary to induce expression of the SASP.

One important question that remains is how does p16 loss mechanistically affect SASP gene expression? p16 is a critical cell cycle regulator, and its suppression both enhances proliferation and allows for senescence bypass [8–10, 12, 82, 83]. Decreased LMNB1 expression plays an important role in the establishment of the SASP [84]. Recent publications demonstrate that decreased expression of LMNB1 and the consequential decrease of nuclear integrity leads to the accumulation of cytoplasmic chromatin fragments (CCFs) that in turn activate the cGAS-STING signaling pathway to drive the SASP [36–38, 85, 86]. Importantly, we found that knockdown of p16 decreased SASP gene expression, which was not a consequence of increased LMNB1 expression (Figure 2). These data suggest that the observed changes in SASP gene expression are not due to downregulation of cGAS-STING signaling, which is directly affected by LMNB1 and nuclear integrity [36]. Notably, we found that four out of the six tumor types analyzed here have decreased cytosolic DNA sensing pathway signaling in p16-low vs. p16-high tumors (Supplementary Figure 4B); however, no correlation was found between *CDKN2A* and *LMNB1* mRNA expression (Figure 4C), suggesting that additional mechanisms are at play in tumors with low p16 expression. It is important to note that besides loss of nuclear integrity, there are other phenomena (mitochondrial DNA leakage, upregulation of LINE-1, or increased expression of the endonuclease MUS81) that may contribute to an impaired cytosolic DNA sensing pathway (reviewed in [87]). Loss of *CDKN2A* is often due to deletion or hypermethylation of the locus [70]. Interestingly, previous work has suggested that melanomas with low *MTAP* have decreased cGAS-STING signaling [88], and *MTAP* is often co-deleted/silenced with *CDKN2A* [89]. Therefore, multiple mechanisms may exist in tumors with loss of this locus to suppress SASP gene expression and/or modulate the tumor microenvironment. Additionally, p16 can negatively regulate *TP53* (encoding for p53) at the transcriptional level and also at the protein level by increasing Mdm2-dependent degradation of p53 [90, 91]. As p53 is a negative regulator of the SASP [92], it is possible that the observed decrease in SASP expression upon p16 suppression is due to negative regulation of p53. Future studies are needed to determine the exact mechanism by which p16 suppression decreases SASP gene expression. Moreover, determining whether these transcription changes in SASP gene expression are a direct or indirect effect of low p16 expression will be of great importance.

We and others have shown that in addition to its canonical role regulating cell cycle progression though the RB pathway, p16 has non-canonical activities that regulate other important aspects of cellular physiology such as nucleotide metabolism, reactive oxygen species, and miRNAs among others [93, 94]. In this regard, some studies have found that pharmacological inhibition of canonical downstream targets of p16, namely CDK4/6, leads to an induction of SASP factors, recruitment of antitumor immune cells, and senescence [95–98], suggesting that p16-loss-mediated regulation of SASP expression and the tumor immune microenvironment may be due to non-canonical (RB-independent) mechanisms. On the contrary, other authors suggest that inhibition of CDK4/6 alone does not induce a SASP and immunologic responses [99]. Additional studies are needed to delineate the exact mechanism whereby suppression of p16 decreases SASP and verify that this occurs in an RB-independent, non-canonical pathway.

Our data suggest that multiple tumor types with low *CDKN2A* (i.e., p16-low) expression have decreased SASP factor expression (Figure 4). A potential caveat of our study is that the SASP factor list we have used for our analysis is broad, and some factors are related to secretion in exosomes [54]. Use of a broad, validated list of SASP factors, while imperfect, allowed us to analyze the TCGA data in a more unbiased fashion versus manually curating a list. Additionally, we demonstrated that knockdown of p16 in BRAF^V600E^-induced senescence *in vitro* corresponds with a decrease in different SASP factors including inflammatory factors, growth factors, metalloproteinases, and ligands (Figure 1I, 1J and Supplementary Figure 1I). Nonetheless, our study shows that low *CDKN2A* expression correlates with downregulation of a distinctive SASP profile depending on the tissue of origin (Figure 4A and Supplementary Table 2). This observation is consistent with previous studies suggesting that the SASP composition is temporally dynamic and context- and senescence inducer-dependent [54, 55, 100]. Characterization of the different SASP profiles and their unique dynamics will be critical not only to assess the senescent cell burden, but also to develop specific and personalized senescence- and SASP-targeted therapies. Moreover, it will be important to determine whether these tumor-specific SASP signatures alter the clinical course of each tumor type or response to therapy. This study is focused on the transcriptional expression regulation of the SASP upon loss of p16; however, additional studies investigating whether these findings impact the translation and secretion of the SASP, especially within exosomes, are warranted. Since suppression of p16 can lead to senescence bypass and promote tumorigenesis [8–10, 12, 82, 83], obtaining profiles of the SASP factors related to this process may help treat the ~50% of all human tumors with low p16 expression [59].

It is well-established that the SASP has pleiotropic, context-dependent effects that both promote tumor progression, but also enhance anti-tumor immunity (reviewed in [101]). For example, IL6 promotes chronic inflammation and tumorigenesis [102]. However, recent studies suggest that IL6 can also enhance anti-tumor immunity by resculpting T cell-mediated immune responses [102]. Likewise, other SASP factors, such as IL1a, IL1b, and TNF have this dual role where they can both promote inflammation and tumorigenesis or impair malignant transformation of benign nevi [103]. Using data from TCGA, we found decreased ‘Antigen Processing and Presentation’ signaling [i.e., the ability of antigen presenting cells to present antigens on major histocompatibility complexes (MHCs) to T-cells] in some patients with low *CDKN2A* expression (Supplementary Figure 4B). Consistent with our observation, it has been shown that OIS primary human melanocytes upregulate the MHC class II apparatus to induce T-cell proliferation and that melanoma patients that sustain this feature have a favorable disease outcome [104]. Additionally, suppression of p16 activity has been associated with immune deserts, immune escape, and low cytolytic activity in melanoma and pancreatic adenocarcinoma [105–107]. Thus, it is possible that in the context of certain tumor types such as those studied here, the decreased expression of SASP factors observed upon p16 knockdown or in *CDKN2A*-low patients may contribute to abrogation of senescence surveillance by immune cells [48, 108], thereby promoting tumorigenesis. Interestingly, a recent publication shows that depletion of p16 in tumor cells abrogates the cancer immune response and promotes immune checkpoint blockade resistance [109]. Here, we did not observe a difference in immune cell infiltration between p16-low and p16-high patient samples (Supplementary Figure 4C), which may suggest that the activity of the immune cells is altered. Future experiments will determine whether suppression of p16 leads to decreased immune surveillance and the mechanism whereby this occurs.

Although loss of p16 is one of the most common events in cancer (~50% of all human cancers), there are currently no approved targeted therapies [93]. Additionally, we and others have shown that suppression of p16 has roles outside the cell cycle that would not be affected with current therapies undergoing clinical trials targeting CDK4/6 [93]. Therefore, finding downstream targetable pathways may be beneficial for this large subset of patients. For instance, we previously showed that inhibition of nucleotide metabolism through suppression of mTORC1 or the pentose phosphate pathway enzyme Ribose 5-Phosphate Isomerase A (RPIA) induces senescence specifically in p16-low cancers [8]. Here we found that suppression of p16 leads to decreased SASP expression. Therefore, induction of senescence in p16-low cancers may be a valuable strategy to inhibit the cell cycle while not activating the potential deleterious effects of the SASP.

In summary, we found that suppression of p16 decreases expression of multiple SASP genes, which cannot be explained by inhibition of senescence. We found that this phenomenon also occurs in p16-wildtype tumor cells upon p16 knockdown, and there is a decrease in the SASP gene signature in multiple tumor types that are associated with low p16 expression. Understanding whether p16 regulates SASP expression is critical to understand the complex relationship between cellular senescence, the immune system, and the cell cycle, three key players in cancer regulation.

## MATERIALS AND METHODS

### Cell lines

Normal diploid IMR90 human fibroblasts were obtained from ATCC (CCL-186) and cultured according to the ATCC protocol in DMEM (Corning, cat#10-017-CV) supplemented with 5% FBS (VWR, cat#97068-085), L-glutamine (Corning, cat#25-015-CI), non-essential amino acids (Corning, cat#25-025-CI), sodium pyruvate (Corning, cat#25-000-CI), and sodium bicarbonate (Corning, cat#25-035-CI). Cells were cultured under physiological oxygen conditions (2% O_2_) and 5% CO_2_. Normal skin fibroblasts derived from a melanoma patient Hs 895.Sk were obtained from ATCC (CRL-7636) and cultured in DMEM (Corning, cat#10-013-CV) supplemented with 10% FBS (VWR, cat#97068-085). Experiments were performed on IMR90 between population doubling #25-35 and in Hs 895.Sk between population doubling #4-10. Melanoma cell lines SKMel28, Hs 600.T, and RPMI-7951, were obtained from ATCC (HTB-72, CRL-7368, and HTB-66, respectively). SKMel28 and Hs 600.T as well as the lentiviral and retroviral packaging cells (293FT and Phoenix, respectively) were cultured in DMEM (Corning, cat#10-013-CV) supplemented with 10% FBS, while RPMI-7951 were cultured in EMEM (ATCC, cat#30-2003) supplemented with 10% FBS. Hs 895.Sk and cancer cell lines were cultured under atmospheric oxygen (~20%) and 5% CO_2_. All cell lines were cultured in MycoZap (Lonza, cat#VZA-2032) and were tested for mycoplasma every two months as described in [110]. All tumor cell lines express wildtype *CDKN2A* according to TCGA [111, 112].

### Lentiviral and retroviral packaging and infection

pBabe BRAF^V600E^ (Addgene cat#15269), pBabe HRAS^G12V^ (Addgene cat#9051), and pBabe empty control (Addgene cat#1764) vectors were packaged into retroviral particles using the BBS/calcium chloride method as previously described in [8]. pLKO.1-shp16 #1 (TRCN0000010482), pLKO.1-shp16 #2 (TRCN0000039751), and pLKO.1-shGFP control (Addgene, cat#30323) vectors were packaged using the ViraPower Kit (Invitrogen, cat# K497500). Experimental timelines for IMR90 and Hs 895.Sk are delineated in Supplementary Figure 1A and Supplementary Figure 2A. Briefly, cells were infected with pBabe empty vector control, pBabe BRAF^V600E^, or pBabe HRAS^G12V^ retroviral particles, and 24 hours later cells were infected with a second round of corresponding retroviral particles. Cells were infected with pLKO.1-shp16 or pLKO.1-shGFP when indicated in Supplementary Figure 1A and Supplementary Figure 2A. As noted in Figure Legends, empty vector pBabe and/or pLKO.1-shGFP were used as controls for all experiments to limit possible effects from expression of viral vectors. Cells were selected with 1μg/mL puromycin for single infections or 3μg/mL for double infections until the end of the experimental procedure.

### Etoposide-induced senescence

SKMel28 melanoma cells (p16-wildtype) with stable p16 knockdown (using shp16 hairpin #1) or control (shGFP) were treated with either DMSO or 1μM etoposide (Cayman Chemical, cat#12092) for 6 days (drug replacement every 2 days).

### RT-qPCR

Total RNA was extracted from cells with Trizol (Ambion, cat#15596018) and DNase treated, cleaned, and concentrated using Zymo columns (Zymo Research, cat#R1013) following the manufacturer’s instructions. Optical density values for RNA were measured using NanoDrop One (Thermo Scientific) to confirm an A260 and A280 ratios above 1.9. Relative expression of target genes was analyzed using the QuantStudio 3 Real-Time PCR System (Thermo Fisher Scientific) with clear 96-well plates (Greiner Bio-One, cat#652240). Primers were designed using the Integrated DNA Technologies (IDT) web tool (Supplementary Table 4). A total of 50ng of RNA was used for One-Step qPCR (Quanta BioSciences, cat# 95089-200) following the manufacturer’s instructions in a final volume of 10μL. Conditions for amplification were: 10 min at 48° C, 5 min at 95° C, 40 cycles of 10 sec at 95° C and 7 sec at 62° C. The assay ended with a melting curve program: 15 sec at 95° C, 1 min at 70° C, then ramping to 95° C while continuously monitoring fluorescence. Each sample was assessed in triplicate. Relative quantification was determined to multiple reference genes (*MRPL19*, *PSMC4*, and *PUM1*) to ensure reproducibility using the delta-delta CT method.

### Western blotting

Cell lysates were collected in 1X sample buffer (2% SDS, 10% glycerol, 0.01% bromophenol blue, 62.5mM Tris, pH=6.8, 0.1M DTT) and boiled (10 min, 95° C). Protein concentration was determined using Bradford assay (Bio-Rad, cat#5000006). Proteins were resolved using SDS-PAGE gels and transferred to nitrocellulose membranes (GE Healthcare Life Sciences, cat#10600001) as previously described [8]. Antibodies used include: anti-BRAF (Santa Cruz Biotechnology, cat#sc-5284, 1:1000), anti-RAS (BD Sciences, cat#610001, 1:1000), anti-p16 (Abcam, cat#ab108349, 1:1000), anti-p21 (Abcam cat#ab109199, 1:1000), anti-cyclin A2 (Abcam cat#ab181591, 1:2000), anti-vinculin (Sigma-Aldrich cat#V9131, 1:1000), β-Actin (Sigma-Aldrich, cat#A1978, 1:10000), anti-mouse HRP (Cell Signaling Technology, cat#cst7076, 1:10,000), and anti-rabbit HRP (Cell Signaling Technology, cat#cst7074, 1:5000).

### Senescence and proliferation assays

SA-β-Gal staining was performed as previously described [113]. Cells were fixed in 2% formaldehyde/0.2% glutaraldehyde in PBS (5 min) and stained (40 mM Na_2_HPO_4_, 150 mM NaCl, 2 mM MgCl_2_, 5mMK_3_Fe(CN)_6_, 5 mM K_4_Fe(CN)_6_, and 1 mg/ml X-gal) overnight at 37° C in a non-CO_2_ incubator. Images were acquired at room temperature using an inverted microscope (Nikon Eclipse Ts2) with a 20X/0.40 objective (Nikon LWD) equipped with a camera (Nikon DS-Fi3). Each sample was assessed in triplicate and at least 100 cells per well were counted (>300 cells per experiment). For colony formation, an equal number of cells were seeded in 6-well plates (for IMR90s) and 12-well plates (for SKMel28) and cultured for an additional 1-2 weeks. Colony formation was visualized by fixing cells in 1% paraformaldehyde (5 min) and staining with 0.05% crystal violet (20 min). Wells were de-stained in 500mL 10% acetic acid (10 min). Absorbance (590nm) was measured using a spectrophotometer (Spectra Max 190). Each sample was assessed in triplicate.

### Differential expression analysis

Preprocessed and processed RNA-Seq data from primary tumors of skin cutaneous melanoma (SKCM), pancreatic adenocarcinoma (PAAD), colorectal adenocarcinoma (COADREAD), mesothelioma (MESO), bladder urothelial carcinoma (BLCA), and glioblastoma multiforme (GBM) TCGA data sets were downloaded from BROAD GDAC Firehose on June 22, 2020 (SKCM, COADREAD, and PAAD) and October 6, 2020 (MESO, BLCA, GBM) [Broad Institute TCGA Genome Data Analysis Center (2016): Firehose 2016_01_28 run. Broad Institute of MIT and Harvard. doi:10.7908/C11G0KM9)]. Processed rnaseqv2 files containing normalized RSEM expression values for each gene in each patient were used to determine the first and third quartile of *CDKN2A* expression for each tumor type separately (Table 1). Quartile values were used to classify patients into p16-low (*CDKN2A* expression ≤ Q1) and p16-high (*CDKN2A* expression ≥ Q3) groups. Differential expression analysis between p16-low and p16-high patients for each tumor type was performed using the preprocessed raw-counts files in R-CRAN (R-3.6.3) and the DESeq2 package.

### Gene set enrichment analysis (GSEA)

Genes were ranked according to the fold-change and p-value obtained in the differential expression analysis between p16-low vs. p16-high as follows: -log_10_(p-value) x sign (log_2_ fold change). Pre-ranked files were built for each tumor type separately in R-CRAN (R-3.6.3) and used to run pre-ranked GSEA (javaGSEA desktop application) for KEGG and Reactome under predefined parameters (1000 permutations, weighted enrichment statistic, excluding sets larger than 500 and smaller than 15 and using meandiv normalization mode, there were no repeated genes thus collapse mode was not used). Following GSEA documentation indications, terms were considered significant when the FDR adjusted p-value (q-value) was <0.25 (http://software.broadinstitute.org/gsea/index.jsp). Gene sets for “Casella et al. senescence upregulated genes” (50 genes) and “Casella et al. senescence downregulated genes” (18 genes) were built in GMX files from publicly-available expression senescence signatures [78] and used to run GSEA using previously described pre-ranked files and parameters.

### Statistical analysis

GraphPad Prism version 7.0 was used to perform statistical analysis. The level of significance between two groups was assessed with unpaired t test. For data sets with more than two groups, one-way ANOVA followed by Tukey’s post hoc test was applied. P-values< 0.05 were considered significant. Pearson correlation test in GraphPad Prism version 7.0 was used to assess the correlation between *LMNB1* and *CDKN2A*. The percentages of normal and tumor cells as well as tumor infiltrating lymphocytes, monocytes, and neutrophils for TCGA tumors were obtained from the biospecimen file at BROAD GDAC Firehose on October 6, 2020.

## Supplementary Material

Supplementary Figures

Supplementary Table 1

Supplementary Table 2

Supplementary Table 3

Supplementary Table 4

## References

[1] Collado M, Blasco MA, Serrano M. Cellular senescence in cancer and aging. Cell. 2007; 130:223–33. 10.1016/j.cell.2007.07.003 17662938

[2] Aird KM, Zhang R. Nucleotide metabolism, oncogene-induced senescence and cancer. Cancer Lett. 2015; 356:204–10. 10.1016/j.canlet.2014.01.017 24486217 PMC4115046

[3] Yaswen P, Campisi J. Oncogene-induced senescence pathways weave an intricate tapestry. Cell. 2007; 128:233–34. 10.1016/j.cell.2007.01.005 17254959

[4] Bringold F, Serrano M. Tumor suppressors and oncogenes in cellular senescence. Exp Gerontol. 2000; 35:317–29. 10.1016/s0531-5565(00)00083-8 10832053

[5] Serrano M, Lin AW, McCurrach ME, Beach D, Lowe SW. Oncogenic ras provokes premature cell senescence associated with accumulation of p53 and p16INK4a. Cell. 1997; 88:593–602. 10.1016/s0092-8674(00)81902-9 9054499

[6] Stein GH, Drullinger LF, Soulard A, Dulić V. Differential roles for cyclin-dependent kinase inhibitors p21 and p16 in the mechanisms of senescence and differentiation in human fibroblasts. Mol Cell Biol. 1999; 19:2109–17. 10.1128/mcb.19.3.2109 10022898 PMC84004

[7] Sherr CJ. The INK4a/ARF network in tumour suppression. Nat Rev Mol Cell Biol. 2001; 2:731–37. 10.1038/35096061 11584300

[8] Buj R, Chen CW, Dahl ES, Leon KE, Kuskovsky R, Maglakelidze N, Navaratnarajah M, Zhang G, Doan MT, Jiang H, Zaleski M, Kutzler L, Lacko H, et al. Suppression of p16 induces mTORC1-mediated nucleotide metabolic reprogramming. Cell Rep. 2019; 28:1971–80.e8. 10.1016/j.celrep.2019.07.084 31433975 PMC6716532

[9] Chin L, Pomerantz J, Polsky D, Jacobson M, Cohen C, Cordon-Cardo C, Horner JW 2nd, DePinho RA. Cooperative effects of INK4a and ras in melanoma susceptibility *in vivo*. Genes Dev. 1997; 11:2822–34. 10.1101/gad.11.21.2822 9353252 PMC316663

[10] Dankort D, Filenova E, Collado M, Serrano M, Jones K, McMahon M. A new mouse model to explore the initiation, progression, and therapy of BRAFV600E-induced lung tumors. Genes Dev. 2007; 21:379–84. 10.1101/gad.1516407 17299132 PMC1804325

[11] Goel VK, Ibrahim N, Jiang G, Singhal M, Fee S, Flotte T, Westmoreland S, Haluska FS, Hinds PW, Haluska FG. Melanocytic nevus-like hyperplasia and melanoma in transgenic BRAFV600E mice. Oncogene. 2009; 28:2289–98. 10.1038/onc.2009.95 19398955 PMC3125533

[12] Haferkamp S, Becker TM, Scurr LL, Kefford RF, Rizos H. p16INK4a-induced senescence is disabled by melanoma-associated mutations. Aging Cell. 2008; 7:733–45. 10.1111/j.1474-9726.2008.00422.x 18843795 PMC2582406

[13] Coppé JP, Desprez PY, Krtolica A, Campisi J. The senescence-associated secretory phenotype: the dark side of tumor suppression. Annu Rev Pathol. 2010; 5:99–118. 10.1146/annurev-pathol-121808-102144 20078217 PMC4166495

[14] Acosta JC, Banito A, Wuestefeld T, Georgilis A, Janich P, Morton JP, Athineos D, Kang TW, Lasitschka F, Andrulis M, Pascual G, Morris KJ, Khan S, et al. A complex secretory program orchestrated by the inflammasome controls paracrine senescence. Nat Cell Biol. 2013; 15:978–90. 10.1038/ncb2784 23770676 PMC3732483

[15] Baker DJ, Wijshake T, Tchkonia T, LeBrasseur NK, Childs BG, van de Sluis B, Kirkland JL, van Deursen JM. Clearance of p16Ink4a-positive senescent cells delays ageing-associated disorders. Nature. 2011; 479:232–36. 10.1038/nature10600 22048312 PMC3468323

[16] Coppé JP, Patil CK, Rodier F, Sun Y, Muñoz DP, Goldstein J, Nelson PS, Desprez PY, Campisi J. Senescence-associated secretory phenotypes reveal cell-nonautonomous functions of oncogenic RAS and the p53 tumor suppressor. PLoS Biol. 2008; 6:2853–68. 10.1371/journal.pbio.0060301 19053174 PMC2592359

[17] Laberge RM, Sun Y, Orjalo AV, Patil CK, Freund A, Zhou L, Curran SC, Davalos AR, Wilson-Edell KA, Liu S, Limbad C, Demaria M, Li P, et al. MTOR regulates the pro-tumorigenic senescence-associated secretory phenotype by promoting IL1A translation. Nat Cell Biol. 2015; 17:1049–61. 10.1038/ncb3195 26147250 PMC4691706

[18] Takasugi M, Okada R, Takahashi A, Virya Chen D, Watanabe S, Hara E. Small extracellular vesicles secreted from senescent cells promote cancer cell proliferation through EphA2. Nat Commun. 2017; 8:15729. 10.1038/ncomms15728 28585531 PMC5467215

[19] Acosta JC, O’Loghlen A, Banito A, Guijarro MV, Augert A, Raguz S, Fumagalli M, Da Costa M, Brown C, Popov N, Takatsu Y, Melamed J, d’Adda di Fagagna F, et al. Chemokine signaling via the CXCR2 receptor reinforces senescence. Cell. 2008; 133:1006–18. 10.1016/j.cell.2008.03.038 18555777

[20] Freund A, Patil CK, Campisi J. p38MAPK is a novel DNA damage response-independent regulator of the senescence-associated secretory phenotype. EMBO J. 2011; 30:1536–48. 10.1038/emboj.2011.69 21399611 PMC3102277

[21] Kang C, Xu Q, Martin TD, Li MZ, Demaria M, Aron L, Lu T, Yankner BA, Campisi J, Elledge SJ. The DNA damage response induces inflammation and senescence by inhibiting autophagy of GATA4. Science. 2015; 349:aaa5612. 10.1126/science.aaa5612 26404840 PMC4942138

[22] Kuilman T, Michaloglou C, Vredeveld LC, Douma S, van Doorn R, Desmet CJ, Aarden LA, Mooi WJ, Peeper DS. Oncogene-induced senescence relayed by an interleukin-dependent inflammatory network. Cell. 2008; 133:1019–31. 10.1016/j.cell.2008.03.039 18555778

[23] Malaquin N, Olivier MA, Martinez A, Nadeau S, Sawchyn C, Coppé JP, Cardin G, Mallette FA, Campisi J, Rodier F. Non-canonical ATM/MRN activities temporally define the senescence secretory program. EMBO Rep. 2020; 21:e50718. 10.15252/embr.202050718 32785991 PMC7534619

[24] Rodier F, Coppé JP, Patil CK, Hoeijmakers WA, Muñoz DP, Raza SR, Freund A, Campeau E, Davalos AR, Campisi J. Persistent DNA damage signalling triggers senescence-associated inflammatory cytokine secretion. Nat Cell Biol. 2009; 11:973–79. 10.1038/ncb1909 19597488 PMC2743561

[25] Zhao J, Zhang L, Lu A, Han Y, Colangelo D, Bukata C, Scibetta A, Yousefzadeh MJ, Li X, Gurkar AU, McGowan SJ, Angelini L, O’Kelly R, et al. ATM is a key driver of NF-κB-dependent DNA-damage-induced senescence, stem cell dysfunction and aging. Aging (Albany NY). 2020; 12:4688–710. 10.18632/aging.102863 32201398 PMC7138542

[26] Aird KM, Iwasaki O, Kossenkov AV, Tanizawa H, Fatkhutdinov N, Bitler BG, Le L, Alicea G, Yang TL, Johnson FB, Noma KI, Zhang R. HMGB2 orchestrates the chromatin landscape of senescence-associated secretory phenotype gene loci. J Cell Biol. 2016; 215:325–34. 10.1083/jcb.201608026 27799366 PMC5100296

[27] Capell BC, Drake AM, Zhu J, Shah PP, Dou Z, Dorsey J, Simola DF, Donahue G, Sammons M, Rai TS, Natale C, Ridky TW, Adams PD, Berger SL. MLL1 is essential for the senescence-associated secretory phenotype. Genes Dev. 2016; 30:321–36. 10.1101/gad.271882.115 26833731 PMC4743061

[28] Chen H, Ruiz PD, McKimpson WM, Novikov L, Kitsis RN, Gamble MJ. MacroH2A1 and ATM play opposing roles in paracrine senescence and the senescence-associated secretory phenotype. Mol Cell. 2015; 59:719–31. 10.1016/j.molcel.2015.07.011 26300260 PMC4548812

[29] Hayakawa T, Iwai M, Aoki S, Takimoto K, Maruyama M, Maruyama W, Motoyama N. SIRT1 suppresses the senescence-associated secretory phenotype through epigenetic gene regulation. PLoS One. 2015; 10:e0116480. 10.1371/journal.pone.0116480 25635860 PMC4312089

[30] Leon KE, Aird KM. Jumonji C demethylases in cellular senescence. Genes (Basel). 2019; 10:33. 10.3390/genes10010033 30634491 PMC6356615

[31] Zhang B, Long Q, Wu S, Song S, Xu Q, Han L, Qian M, Ren X, Jiang J, Fu Q, Guo J, Zhang X, Chang X, et al. KDM4 Orchestrates Epigenomic Remodeling of Senescent Cells and Potentiates the Senescence-Associated Secretory Phenotype. bioRxiv. 2020. 10.1101/2020.08.03.235465 PMC8277122 34263179

[32] Leon KE, Buj R, Lesko E, Dahl ES, Chen C-W, Imamura Y, Kossenkov AV, Hobbs RP, Aird KM. DOT1L modulates the senescence-associated secretory phenotype through epigenetic regulation of IL1A. bioRxiv. 2020. 10.1101/2020.08.21.258020 PMC8160577 34037658

[33] Herranz N, Gallage S, Mellone M, Wuestefeld T, Klotz S, Hanley CJ, Raguz S, Acosta JC, Innes AJ, Banito A, Georgilis A, Montoya A, Wolter K, et al. mTOR regulates MAPKAPK2 translation to control the senescence-associated secretory phenotype. Nat Cell Biol. 2015; 17:1205–17. 10.1038/ncb3225 26280535 PMC4589897

[34] Freund A, Laberge RM, Demaria M, Campisi J. Lamin B1 loss is a senescence-associated biomarker. Mol Biol Cell. 2012; 23:2066–75. 10.1091/mbc.E11-10-0884 22496421 PMC3364172

[35] Shimi T, Butin-Israeli V, Adam SA, Hamanaka RB, Goldman AE, Lucas CA, Shumaker DK, Kosak ST, Chandel NS, Goldman RD. The role of nuclear lamin B1 in cell proliferation and senescence. Genes Dev. 2011; 25:2579–93. 10.1101/gad.179515.111 22155925 PMC3248680

[36] Dou Z, Ghosh K, Vizioli MG, Zhu J, Sen P, Wangensteen KJ, Simithy J, Lan Y, Lin Y, Zhou Z, Capell BC, Xu C, Xu M, et al. Cytoplasmic chromatin triggers inflammation in senescence and cancer. Nature. 2017; 550:402–06. 10.1038/nature24050 28976970 PMC5850938

[37] Ivanov A, Pawlikowski J, Manoharan I, van Tuyn J, Nelson DM, Rai TS, Shah PP, Hewitt G, Korolchuk VI, Passos JF, Wu H, Berger SL, Adams PD. Lysosome-mediated processing of chromatin in senescence. J Cell Biol. 2013; 202:129–43. 10.1083/jcb.201212110 23816621 PMC3704985

[38] Glück S, Guey B, Gulen MF, Wolter K, Kang TW, Schmacke NA, Bridgeman A, Rehwinkel J, Zender L, Ablasser A. Innate immune sensing of cytosolic chromatin fragments through cGAS promotes senescence. Nat Cell Biol. 2017; 19:1061–70. 10.1038/ncb3586 28759028 PMC5826565

[39] Yang H, Wang H, Ren J, Chen Q, Chen ZJ. cGAS is essential for cellular senescence. Proc Natl Acad Sci USA. 2017; 114:E4612–20. 10.1073/pnas.1705499114 28533362 PMC5468617

[40] Fane M, Weeraratna AT. How the ageing microenvironment influences tumour progression. Nat Rev Cancer. 2020; 20:89–106. 10.1038/s41568-019-0222-9 31836838 PMC7377404

[41] Da Silva-Álvarez S, Guerra-Varela J, Sobrido-Cameán D, Quelle A, Barreiro-Iglesias A, Sánchez L, Collado M. Cell senescence contributes to tissue regeneration in zebrafish. Aging Cell. 2020; 19:e13052. 10.1111/acel.13052 31670873 PMC6974711

[42] Demaria M, Ohtani N, Youssef SA, Rodier F, Toussaint W, Mitchell JR, Laberge RM, Vijg J, Van Steeg H, Dollé ME, Hoeijmakers JH, de Bruin A, Hara E, Campisi J. An essential role for senescent cells in optimal wound healing through secretion of PDGF-AA. Dev Cell. 2014; 31:722–33. 10.1016/j.devcel.2014.11.012 25499914 PMC4349629

[43] Yun MH, Davaapil H, Brockes JP. Recurrent turnover of senescent cells during regeneration of a complex structure. Elife. 2015; 4:e05505. 10.7554/eLife.05505 25942455 PMC4434796

[44] Muñoz-Espín D, Cañamero M, Maraver A, Gómez-López G, Contreras J, Murillo-Cuesta S, Rodríguez-Baeza A, Varela-Nieto I, Ruberte J, Collado M, Serrano M. Programmed cell senescence during mammalian embryonic development. Cell. 2013; 155:1104–18. 10.1016/j.cell.2013.10.019 24238962

[45] Storer M, Mas A, Robert-Moreno A, Pecoraro M, Ortells MC, Di Giacomo V, Yosef R, Pilpel N, Krizhanovsky V, Sharpe J, Keyes WM. Senescence is a developmental mechanism that contributes to embryonic growth and patterning. Cell. 2013; 155:1119–30. 10.1016/j.cell.2013.10.041 24238961

[46] Eggert T, Wolter K, Ji J, Ma C, Yevsa T, Klotz S, Medina-Echeverz J, Longerich T, Forgues M, Reisinger F, Heikenwalder M, Wang XW, Zender L, Greten TF. Distinct functions of senescence-associated immune responses in liver tumor surveillance and tumor progression. Cancer Cell. 2016; 30:533–47. 10.1016/j.ccell.2016.09.003 27728804 PMC7789819

[47] Iannello A, Thompson TW, Ardolino M, Lowe SW, Raulet DH. P53-dependent chemokine production by senescent tumor cells supports NKG2D-dependent tumor elimination by natural killer cells. J Exp Med. 2013; 210:2057–69. 10.1084/jem.20130783 24043758 PMC3782044

[48] Kang TW, Yevsa T, Woller N, Hoenicke L, Wuestefeld T, Dauch D, Hohmeyer A, Gereke M, Rudalska R, Potapova A, Iken M, Vucur M, Weiss S, et al. Senescence surveillance of pre-malignant hepatocytes limits liver cancer development. Nature. 2011; 479:547–51. 10.1038/nature10599 22080947

[49] Bavik C, Coleman I, Dean JP, Knudsen B, Plymate S, Nelson PS. The gene expression program of prostate fibroblast senescence modulates neoplastic epithelial cell proliferation through paracrine mechanisms. Cancer Res. 2006; 66:794–802. 10.1158/0008-5472.CAN-05-1716 16424011

[50] Canino C, Mori F, Cambria A, Diamantini A, Germoni S, Alessandrini G, Borsellino G, Galati R, Battistini L, Blandino R, Facciolo F, Citro G, Strano S, et al. SASP mediates chemoresistance and tumor-initiating-activity of mesothelioma cells. Oncogene. 2012; 31:3148–63. 10.1038/onc.2011.485 22020330

[51] Coppé JP, Kauser K, Campisi J, Beauséjour CM. Secretion of vascular endothelial growth factor by primary human fibroblasts at senescence. J Biol Chem. 2006; 281:29568–74. 10.1074/jbc.M603307200 16880208

[52] Yang F, Tuxhorn JA, Ressler SJ, McAlhany SJ, Dang TD, Rowley DR. Stromal expression of connective tissue growth factor promotes angiogenesis and prostate cancer tumorigenesis. Cancer Res. 2005; 65:8887–95. 10.1158/0008-5472.CAN-05-1702 16204060

[53] Yoshimoto S, Loo TM, Atarashi K, Kanda H, Sato S, Oyadomari S, Iwakura Y, Oshima K, Morita H, Hattori M, Honda K, Ishikawa Y, Hara E, Ohtani N. Obesity-induced gut microbial metabolite promotes liver cancer through senescence secretome. Nature. 2013; 499:97–101. 10.1038/nature12347 23803760

[54] Basisty N, Kale A, Jeon OH, Kuehnemann C, Payne T, Rao C, Holtz A, Shah S, Sharma V, Ferrucci L, Campisi J, Schilling B. A proteomic atlas of senescence-associated secretomes for aging biomarker development. PLoS Biol. 2020; 18:e3000599. 10.1371/journal.pbio.3000599 31945054 PMC6964821

[55] Hernandez-Segura A, de Jong TV, Melov S, Guryev V, Campisi J, Demaria M. Unmasking transcriptional heterogeneity in senescent cells. Curr Biol. 2017; 27:2652–60.e4. 10.1016/j.cub.2017.07.033 28844647 PMC5788810

[56] Toso A, Revandkar A, Di Mitri D, Guccini I, Proietti M, Sarti M, Pinton S, Zhang J, Kalathur M, Civenni G, Jarrossay D, Montani E, Marini C, et al. Enhancing chemotherapy efficacy in pten-deficient prostate tumors by activating the senescence-associated antitumor immunity. Cell Rep. 2014; 9:75–89. 10.1016/j.celrep.2014.08.044 25263564

[57] Wang B, Kohli J, Demaria M. Senescent Cells in Cancer Therapy: Friends or Foes? Trends Cancer. 2020; 6:838–57. 10.1016/j.trecan.2020.05.004 32482536

[58] Rao SG, Jackson JG. SASP: tumor suppressor or promoter? yes!. Trends Cancer. 2016; 2:676–87. 10.1016/j.trecan.2016.10.001 28741506

[59] Romagosa C, Simonetti S, López-Vicente L, Mazo A, Lleonart ME, Castellvi J, Ramon y Cajal S. p16(Ink4a) overexpression in cancer: a tumor suppressor gene associated with senescence and high-grade tumors. Oncogene. 2011; 30:2087–97. 10.1038/onc.2010.614 21297668

[60] Coppé JP, Rodier F, Patil CK, Freund A, Desprez PY, Campisi J. Tumor suppressor and aging biomarker p16(INK4a) induces cellular senescence without the associated inflammatory secretory phenotype. J Biol Chem. 2011; 286:36396–403. 10.1074/jbc.M111.257071 21880712 PMC3196093

[61] Ortiz-Montero P, Londoño-Vallejo A, Vernot JP. Senescence-associated IL-6 and IL-8 cytokines induce a self- and cross-reinforced senescence/inflammatory milieu strengthening tumorigenic capabilities in the MCF-7 breast cancer cell line. Cell Commun Signal. 2017; 15:17. 10.1186/s12964-017-0172-3 28472950 PMC5418812

[62] Zhu J, Woods D, McMahon M, Bishop JM. Senescence of human fibroblasts induced by oncogenic raf. Genes Dev. 1998; 12:2997–3007. 10.1101/gad.12.19.2997 9765202 PMC317194

[63] Di Micco R, Fumagalli M, Cicalese A, Piccinin S, Gasparini P, Luise C, Schurra C, Garre’ M, Nuciforo PG, Bensimon A, Maestro R, Pelicci PG, d’Adda di Fagagna F. Oncogene-induced senescence is a DNA damage response triggered by DNA hyper-replication. Nature. 2006; 444:638–42. 10.1038/nature05327 17136094

[64] Ogrunc M, Di Micco R, Liontos M, Bombardelli L, Mione M, Fumagalli M, Gorgoulis VG, d’Adda di Fagagna F. Oncogene-induced reactive oxygen species fuel hyperproliferation and DNA damage response activation. Cell Death Differ. 2014; 21:998–1012. 10.1038/cdd.2014.16 24583638 PMC4013514

[65] Aird KM, Zhang G, Li H, Tu Z, Bitler BG, Garipov A, Wu H, Wei Z, Wagner SN, Herlyn M, Zhang R. Suppression of nucleotide metabolism underlies the establishment and maintenance of oncogene-induced senescence. Cell Rep. 2013; 3:1252–65. 10.1016/j.celrep.2013.03.004 23562156 PMC3840499

[66] Chakravarti A, Heydon K, Wu CL, Hammond E, Pollack A, Roach M, Wolkov H, Okunieff P, Cox J, Fontanesi J, Abrams R, Pilepich M, Shipley W, and Radiation Therapy Oncology Group. Loss of p16 expression is of prognostic significance in locally advanced prostate cancer: an analysis from the radiation therapy oncology group protocol 86-10. J Clin Oncol. 2003; 21:3328–34. 10.1200/JCO.2003.12.151 12947069

[67] Straume O, Sviland L, Akslen LA. Loss of nuclear p16 protein expression correlates with increased tumor cell proliferation (Ki-67) and poor prognosis in patients with vertical growth phase melanoma. Clin Cancer Res. 2000; 6:1845–53. 10815907

[68] Weinberger PM, Yu Z, Haffty BG, Kowalski D, Harigopal M, Sasaki C, Rimm DL, Psyrri A. Prognostic significance of p16 protein levels in oropharyngeal squamous cell cancer. Clin Cancer Res. 2004; 10:5684–91. 10.1158/1078-0432.CCR-04-0448 15355894

[69] Witkiewicz AK, Knudsen KE, Dicker AP, Knudsen ES. The meaning of p16(ink4a) expression in tumors: functional significance, clinical associations and future developments. Cell Cycle. 2011; 10:2497–503. 10.4161/cc.10.15.16776 21775818 PMC3685613

[70] Zhao R, Choi BY, Lee MH, Bode AM, Dong Z. Implications of genetic and epigenetic alterations of CDKN2A (p16(INK4a)) in cancer. EBioMedicine. 2016; 8:30–39. 10.1016/j.ebiom.2016.04.017 27428416 PMC4919535

[71] Borczuk AC, Taub RN, Hesdorffer M, Hibshoosh H, Chabot JA, Keohan ML, Alsberry R, Alexis D, Powell CA. P16 loss and mitotic activity predict poor survival in patients with peritoneal Malignant mesothelioma. Clin Cancer Res. 2005; 11:3303–08. 10.1158/1078-0432.CCR-04-1884 15867227

[72] Illei PB, Rusch VW, Zakowski MF, Ladanyi M. Homozygous deletion of CDKN2A and codeletion of the methylthioadenosine phosphorylase gene in the majority of pleural mesotheliomas. Clin Cancer Res. 2003; 9:2108–13. 12796375

[73] Kamiryo T, Tada K, Shiraishi S, Shinojima N, Nakamura H, Kochi M, Kuratsu J, Saya H, Ushio Y. Analysis of homozygous deletion of the p16 gene and correlation with survival in patients with glioblastoma multiforme. J Neurosurg. 2002; 96:815–22. 10.3171/jns.2002.96.5.0815 12005388

[74] Gan X, Lin X, He R, Lin X, Wang H, Yan L, Zhou H, Qin H, Chen G. Prognostic and clinicopathological significance of downregulated p16 expression in patients with bladder cancer: a systematic review and meta-analysis. Dis Markers. 2016; 2016:5259602. 10.1155/2016/5259602 27199504 PMC4854991

[75] Oshima M, Okano K, Muraki S, Haba R, Maeba T, Suzuki Y, Yachida S. Immunohistochemically detected expression of 3 major genes (CDKN2A/p16, TP53, and SMAD4/DPC4) strongly predicts survival in patients with resectable pancreatic cancer. Ann Surg. 2013; 258:336–46. 10.1097/SLA.0b013e3182827a65 23470568

[76] Mihic-Probst D, Mnich CD, Oberholzer PA, Seifert B, Sasse B, Moch H, Dummer R. P16 expression in primary Malignant melanoma is associated with prognosis and lymph node status. Int J Cancer. 2006; 118:2262–68. 10.1002/ijc.21608 16331607

[77] Xing X, Cai W, Shi H, Wang Y, Li M, Jiao J, Chen M. The prognostic value of CDKN2A hypermethylation in colorectal cancer: a meta-analysis. Br J Cancer. 2013; 108:2542–48. 10.1038/bjc.2013.251 23703248 PMC3694241

[78] Casella G, Munk R, Kim KM, Piao Y, De S, Abdelmohsen K, Gorospe M. Transcriptome signature of cellular senescence. Nucleic Acids Res. 2019; 47:11476. 10.1093/nar/gkz879 31612919 PMC6868356

[79] Liu JY, Souroullas GP, Diekman BO, Krishnamurthy J, Hall BM, Sorrentino JA, Parker JS, Sessions GA, Gudkov AV, Sharpless NE. Cells exhibiting strong *p16*^INK4a^ promoter activation *in vivo* display features of senescence. Proc Natl Acad Sci USA. 2019; 116:2603–11. 10.1073/pnas.1818313116 30683717 PMC6377452

[80] Herranz N, Gil J. Mechanisms and functions of cellular senescence. J Clin Invest. 2018; 128:1238–46. 10.1172/JCI95148 29608137 PMC5873888

[81] Meyer P, Maity P, Burkovski A, Schwab J, Müssel C, Singh K, Ferreira FF, Krug L, Maier HJ, Wlaschek M, Wirth T, Kestler HA, Scharffetter-Kochanek K. A model of the onset of the senescence associated secretory phenotype after DNA damage induced senescence. PLoS Comput Biol. 2017; 13:e1005741. 10.1371/journal.pcbi.1005741 29206223 PMC5730191

[82] Damsky W, Micevic G, Meeth K, Muthusamy V, Curley DP, Santhanakrishnan M, Erdelyi I, Platt JT, Huang L, Theodosakis N, Zaidi MR, Tighe S, Davies MA, et al. mTORC1 activation blocks BrafV600E-induced growth arrest but is insufficient for melanoma formation. Cancer Cell. 2015; 27:41–56. 10.1016/j.ccell.2014.11.014 25584893 PMC4295062

[83] Krimpenfort P, Quon KC, Mooi WJ, Loonstra A, Berns A. Loss of p16Ink4a confers susceptibility to metastatic melanoma in mice. Nature. 2001; 413:83–86. 10.1038/35092584 11544530

[84] Shah PP, Donahue G, Otte GL, Capell BC, Nelson DM, Cao K, Aggarwala V, Cruickshanks HA, Rai TS, McBryan T, Gregory BD, Adams PD, Berger SL. Lamin B1 depletion in senescent cells triggers large-scale changes in gene expression and the chromatin landscape. Genes Dev. 2013; 27:1787–99. 10.1101/gad.223834.113 23934658 PMC3759695

[85] Hari P, Millar FR, Tarrats N, Birch J, Quintanilla A, Rink CJ, Fernández-Duran I, Muir M, Finch AJ, Brunton VG, Passos JF, Morton JP, Boulter L, Acosta JC. The innate immune sensor toll-like receptor 2 controls the senescence-associated secretory phenotype. Sci Adv. 2019; 5:eaaw0254. 10.1126/sciadv.aaw0254 31183403 PMC6551188

[86] Takahashi A, Loo TM, Okada R, Kamachi F, Watanabe Y, Wakita M, Watanabe S, Kawamoto S, Miyata K, Barber GN, Ohtani N, Hara E. Downregulation of cytoplasmic DNases is implicated in cytoplasmic DNA accumulation and SASP in senescent cells. Nat Commun. 2018; 9:1249. 10.1038/s41467-018-03555-8 29593264 PMC5871854

[87] Gao M, He Y, Tang H, Chen X, Liu S, Tao Y. cGAS/STING: novel perspectives of the classic pathway. Molecular Biomedicine. 2020; 1:7. 10.1186/s43556-020-00006-z PMC8603984 35006429

[88] Kim H, Kim H, Feng Y, Li Y, Tamiya H, Tocci S, Ronai ZA. PRMT5 control of cGAS/STING and NLRC5 pathways defines melanoma response to antitumor immunity. Sci Transl Med. 2020; 12:eaaz5683. 10.1126/scitranslmed.aaz5683 32641491 PMC7508354

[89] Zhang H, Chen ZH, Savarese TM. Codeletion of the genes for p16INK4, methylthioadenosine phosphorylase, interferon-alpha1, interferon-beta1, and other 9p21 markers in human Malignant cell lines. Cancer Genet Cytogenet. 1996; 86:22–28. 10.1016/0165-4608(95)00157-3 8616780

[90] Huschtscha LI, Moore JD, Noble JR, Campbell HG, Royds JA, Braithwaite AW, Reddel RR. Normal human mammary epithelial cells proliferate rapidly in the presence of elevated levels of the tumor suppressors p53 and p21(WAF1/CIP1). J Cell Sci. 2009; 122:2989–95. 10.1242/jcs.044107 19638413

[91] Kubbutat MH, Jones SN, Vousden KH. Regulation of p53 stability by Mdm2. Nature. 1997; 387:299–303. 10.1038/387299a0 9153396

[92] Wiley CD, Schaum N, Alimirah F, Lopez-Dominguez JA, Orjalo AV, Scott G, Desprez PY, Benz C, Davalos AR, Campisi J. Small-molecule MDM2 antagonists attenuate the senescence-associated secretory phenotype. Sci Rep. 2018; 8:2410. 10.1038/s41598-018-20000-4 29402901 PMC5799282

[93] Buj R, Aird KM. P16: cycling off the beaten path. Mol Cell Oncol. 2019; 6:e1677140. 10.1080/23723556.2019.1677140 31692916 PMC6816386

[94] Mirzayans R, Andrais B, Hansen G, Murray D. Role of p16(INK4A) in replicative senescence and DNA damage-induced premature senescence in p53-deficient human cells. Biochem Res Int. 2012; 2012:951574. 10.1155/2012/951574 22924132 PMC3424640

[95] Birnhuber A, Egemnazarov B, Biasin V, Bonyadi Rad E, Wygrecka M, Olschewski H, Kwapiszewska G, Marsh LM. CDK4/6 inhibition enhances pulmonary inflammatory infiltration in bleomycin-induced lung fibrosis. Respir Res. 2020; 21:167. 10.1186/s12931-020-01433-w 32616042 PMC7331186

[96] Goel S, DeCristo MJ, Watt AC, BrinJones H, Sceneay J, Li BB, Khan N, Ubellacker JM, Xie S, Metzger-Filho O, Hoog J, Ellis MJ, Ma CX, et al. CDK4/6 inhibition triggers anti-tumour immunity. Nature. 2017; 548:471–75. 10.1038/nature23465 28813415 PMC5570667

[97] Guan X, LaPak KM, Hennessey RC, Yu CY, Shakya R, Zhang J, Burd CE. Stromal senescence by prolonged CDK4/6 inhibition potentiates tumor growth. Mol Cancer Res. 2017; 15:237–49. 10.1158/1541-7786.MCR-16-0319 28039358 PMC5334447

[98] Yoshida A, Lee EK, Diehl JA. Induction of therapeutic senescence in vemurafenib-resistant melanoma by extended inhibition of CDK4/6. Cancer Res. 2016; 76:2990–3002. 10.1158/0008-5472.CAN-15-2931 26988987 PMC4873417

[99] Ruscetti M, Leibold J, Bott MJ, Fennell M, Kulick A, Salgado NR, Chen CC, Ho YJ, Sanchez-Rivera FJ, Feucht J, Baslan T, Tian S, Chen HA, et al. NK cell-mediated cytotoxicity contributes to tumor control by a cytostatic drug combination. Science. 2018; 362:1416–22. 10.1126/science.aas9090 30573629 PMC6711172

[100] Hoare M, Ito Y, Kang TW, Weekes MP, Matheson NJ, Patten DA, Shetty S, Parry AJ, Menon S, Salama R, Antrobus R, Tomimatsu K, Howat W, et al. NOTCH1 mediates a switch between two distinct secretomes during senescence. Nat Cell Biol. 2016; 18:979–92. 10.1038/ncb3397 27525720 PMC5008465

[101] Faget DV, Ren Q, Stewart SA. Unmasking senescence: context-dependent effects of SASP in cancer. Nat Rev Cancer. 2019; 19:439–53. 10.1038/s41568-019-0156-2 31235879

[102] Fisher DT, Appenheimer MM, Evans SS. The two faces of IL-6 in the tumor microenvironment. Semin Immunol. 2014; 26:38–47. 10.1016/j.smim.2014.01.008 24602448 PMC3970580

[103] Ahmed AA, Nordlind K, Hedblad M, Lagerholm B, Schultzberg M, Lidén S. Interleukin (IL)-1 alpha- and -1 beta-, IL-6-, and tumor necrosis factor-alpha-like immunoreactivities in human common and dysplastic nevocellular nevi and Malignant melanoma. Am J Dermatopathol. 1995; 17:222–29. 10.1097/00000372-199506000-00002 8599429

[104] van Tuyn J, Jaber-Hijazi F, MacKenzie D, Cole JJ, Mann E, Pawlikowski JS, Rai TS, Nelson DM, McBryan T, Ivanov A, Blyth K, Wu H, Milling S, Adams PD. Oncogene-expressing senescent melanocytes up-regulate MHC class II, a candidate melanoma suppressor function. J Invest Dermatol. 2017; 137:2197–207. 10.1016/j.jid.2017.05.030 28647344 PMC5613751

[105] Balli D, Rech AJ, Stanger BZ, Vonderheide RH. Immune cytolytic activity stratifies molecular subsets of human pancreatic cancer. Clin Cancer Res. 2017; 23:3129–38. 10.1158/1078-0432.CCR-16-2128 28007776 PMC12164831

[106] Morrison C, Pabla S, Conroy JM, Nesline MK, Glenn ST, Dressman D, Papanicolau-Sengos A, Burgher B, Andreas J, Giamo V, Qin M, Wang Y, Lenzo FL, et al. Predicting response to checkpoint inhibitors in melanoma beyond PD-L1 and mutational burden. J Immunother Cancer. 2018; 6:32. 10.1186/s40425-018-0344-8 29743104 PMC5944039

[107] Wartenberg M, Cibin S, Zlobec I, Vassella E, Eppenberger-Castori S, Terracciano L, Eichmann MD, Worni M, Gloor B, Perren A, Karamitopoulou E. Integrated genomic and immunophenotypic classification of pancreatic cancer reveals three distinct subtypes with prognostic/predictive significance. Clin Cancer Res. 2018; 24:4444–54. 10.1158/1078-0432.CCR-17-3401 29661773

[108] Hoenicke L, Zender L. Immune surveillance of senescent cells—biological significance in cancer- and non-cancer pathologies. Carcinogenesis. 2012; 33:1123–26. 10.1093/carcin/bgs124 22470164

[109] Brenner E, Schörg BF, Ahmetlić F, Wieder T, Hilke FJ, Simon N, Schroeder C, Demidov G, Riedel T, Fehrenbacher B, Schaller M, Forschner A, Eigentler T, et al. Cancer immune control needs senescence induction by interferon-dependent cell cycle regulator pathways in tumours. Nat Commun. 2020; 11:1335. 10.1038/s41467-020-14987-6 32165639 PMC7067802

[110] Uphoff CC, Drexler HG. Detection of mycoplasma contaminations. Methods Mol Biol. 2005; 290:13–23. 10.1385/1-59259-838-2:013 15361652

[111] Cerami E, Gao J, Dogrusoz U, Gross BE, Sumer SO, Aksoy BA, Jacobsen A, Byrne CJ, Heuer ML, Larsson E, Antipin Y, Reva B, Goldberg AP, et al. The cBio cancer genomics portal: an open platform for exploring multidimensional cancer genomics data. Cancer Discov. 2012; 2:401–04. 10.1158/2159-8290.CD-12-0095 22588877 PMC3956037

[112] Gao J, Aksoy BA, Dogrusoz U, Dresdner G, Gross B, Sumer SO, Sun Y, Jacobsen A, Sinha R, Larsson E, Cerami E, Sander C, Schultz N. Integrative analysis of complex cancer genomics and clinical profiles using the cBioPortal. Sci Signal. 2013; 6:pl1. 10.1126/scisignal.2004088 23550210 PMC4160307

[113] Dimri GP, Lee X, Basile G, Acosta M, Scott G, Roskelley C, Medrano EE, Linskens M, Rubelj I, Pereira-Smith O, et al. A biomarker that identifies senescent human cells in culture and in aging skin *in vivo*. Proc Natl Acad Sci U S A. 1995; 92:9363–7. 10.1073/pnas.92.20.9363 7568133 PMC40985

