# Supplementary material for: Suppression of p16 alleviates the senescence-associated secretory phenotype
Source: Aging (Albany NY). 2021 Feb 6;13(3):3290–312. doi: 10.18632/aging.202640 (PMC7906185; doi:10.18632/aging.202640)
Supplement: Supplementary Figures [file aging-13-202640-s001.pdf]

SUPPLEMENTARY FIGURES

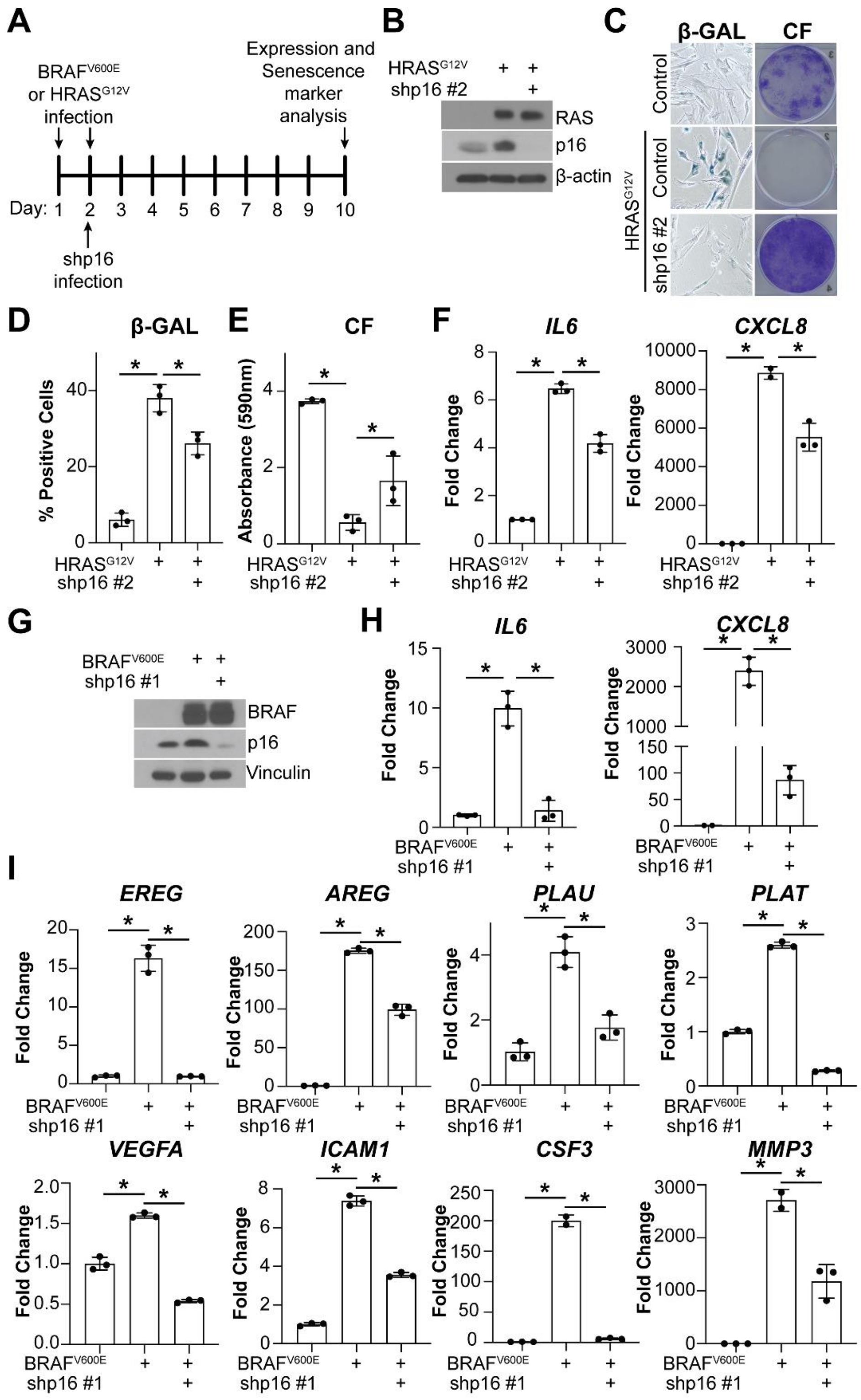

Supplementary Figure 1. Knockdown of p16 using a second hairpin and independent cell line decreases *IL6* and *CXCL8* gene expression in oncogene-induced cells; knockdown of p16 decreases expression of other SASP factors; related to Figure 1. (**A**) Schematic of the experimental procedure for Figure 1 and Supplementary Figure 1. (**B**–**F**) IMR90s expressing HRAS^{G12V} alone or in combination with a shRNA targeting p16 (shp16 hairpin #2). An empty pBabe retroviral and a shRNA targeting GFP lentiviral vector were used

as controls. (**B**) Immunoblot of RAS and p16. β-actin was used as loading control. (**C**) Representative images of senescence-associated β-galactosidase (β-GAL) staining and colony formation (CF). (**D**) Quantification of β-GAL in (**C**). (**E**) Quantification of CF in (**C**). (**F**) *IL6* and *CXCL8* mRNA expression (fold change relative to control mean). (**G**, **H**) Hs 895.Sk cells expressing $BRAF^{V600E}$ alone or in combination with a shRNA targeting p16 (shp16 hairpin #1). An empty pBabe retroviral and a shRNA targeting GFP lentiviral vector were used as controls. (**G**) Immunoblot of BRAF and p16. Vinculin was used as loading control. (**H**) *IL6* and *CXCL8* mRNA expression (fold change relative to control mean). (**I**) IMR90s expressing $BRAF^{V600E}$ alone or in combination with a shRNA targeting p16 (shp16 hairpin #1). An empty pBabe retroviral and a shRNA targeting GFP lentiviral vector were used as controls. mRNA expression of the indicated genes (fold change relative to control mean). For all RT-qPCR, expression of target genes was normalized against multiple reference genes. Data normalized against *MRPL9* are shown. n=3/group and mean±SD. 1 out of 3 experiments is shown. *p<0.05.

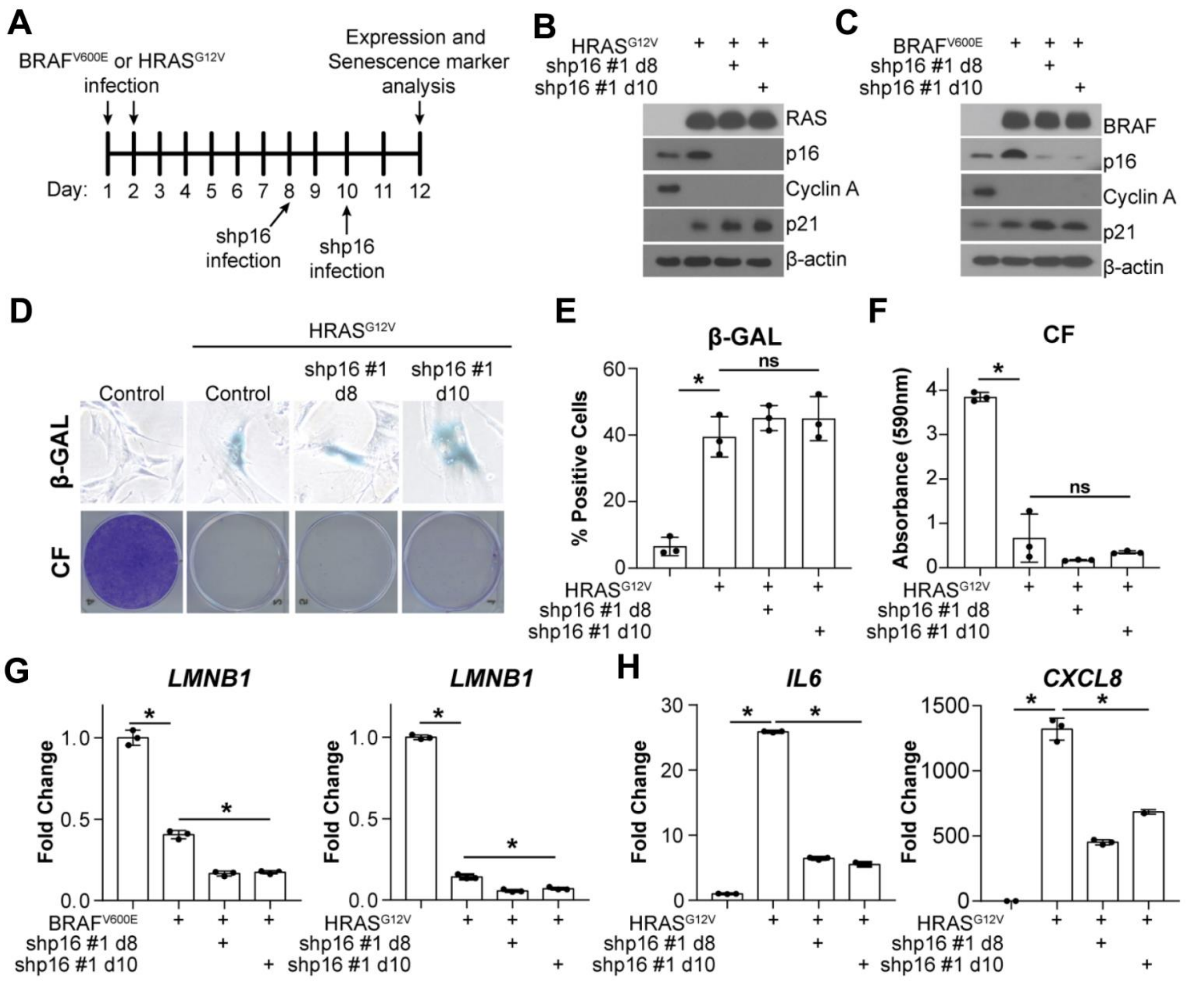

Supplementary Figure 2. Knockdown of p16 at later timepoints decreases *IL6* and *CXCL8* but does affect other senescence markers including *LMNB1*; related to Figure 2. (**A**) Schematic of experimental procedure for Figure 2 and Supplementary Figure 2. (**B–H**) IMR90s expressing either $BRAF^{V600E}$ or $HRAS^{G12V}$ alone or in combination with a shRNA targeting p16 (shp16 hairpin #1). An empty pBabe retroviral and a shRNA targeting GFP lentiviral vector were used as controls. (**B**, **C**) Immunoblot of the indicated proteins. β-actin was used as loading control. (**D**) Representative images of senescence-associated β-galactosidase (β-GAL) staining and colony formation (CF). (**E**) Quantification of β-GAL in (**D**). (**F**) Quantification of CF in (**D**). (**G**) *LMNB1* mRNA expression (fold change relative to control mean). (**H**) *IL6* and *CXCL8* mRNA expression (fold change relative to control mean). For all RT-qPCR, expression of target genes was normalized against multiple reference genes. Data normalized against *MRPL9* are shown. n=3/group and mean±SD. 1 out of 3 experiments is shown. *p<0.05, ns = not significant.

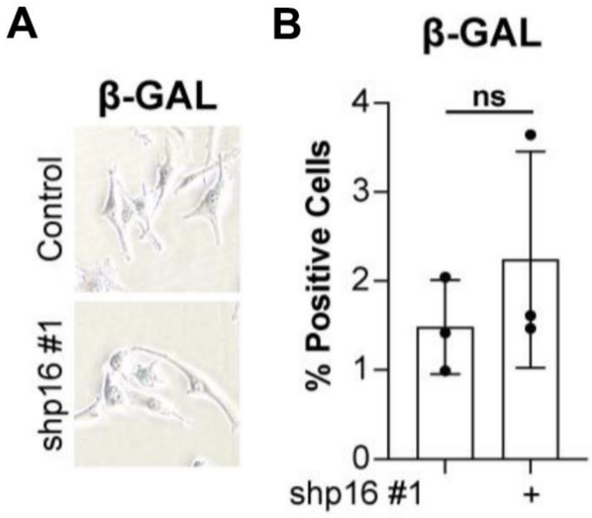

Supplementary Figure 3. Melanoma cells with stable knockdown of p16 do not have less spontaneous senescent cells in culture; related to Figure 3. (**A**, **B**) SKMel28 melanoma cells with stable knockdown of p16 (shp16 hairpin #1). An shRNA targeting GFP lentiviral vector was used as control. (**A**) Representative images of senescence-associated β-galactosidase (β-GAL) staining. (**B**) Quantification of β-GAL in (**A**). ns = not significant.

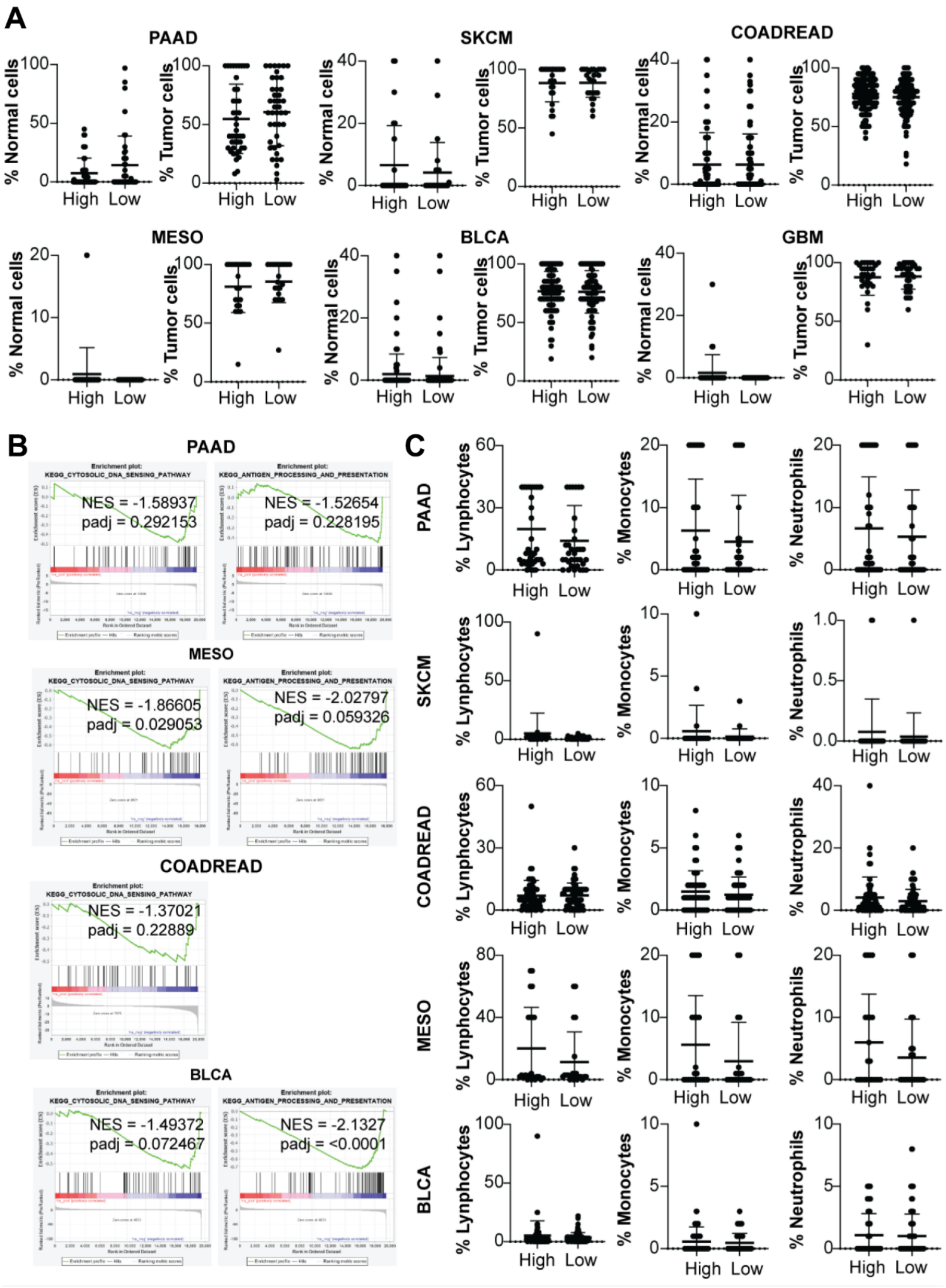

Supplementary Figure 4. Tumors with low CDKN2A expression show decreased inflammation-related signatures not associated with changes in percentage of normal cells or immune cell infiltration; related to Figure 4. (**A**) Percentage of normal and tumor cells seen on an OCT-embedded tissue slide reported by TCGA between CDKN2A-high (i.e., p16-high) and CDKN2A-low (i.e., p16-low) in the indicated tumors. (**B**) Inflammation related negatively enriched terms in p16-low vs. p16-high expressing tumors. (**C**) Percentage of lymphocytes, monocytes, and neutrophils seen on an OCT-embedded tissue slide reported by TCGA between p16-high and p16-low in the indicated tumors. SKCM (skin cutaneous melanoma), PAAD (pancreatic adenocarcinoma), COADREAD (colorectal adenocarcinoma), MESO (mesothelioma), BLCA (bladder urothelial carcinoma), GBM (glioblastoma multiforme), NES (negative enrichment score).
